# Anti-dimerization 56π-electron fullerene adduct bearing bulky functional groups for inverted perovskite solar cells with enhanced interfacial stability

**DOI:** 10.1093/nsr/nwaf466

**Published:** 2025-10-30

**Authors:** Xue Wang, Shenghu Yuan, Shuaihua Lu, Zheng Liang, Shantao Zhang, Rongyao Lv, Xinyu Li, Hongchang Fan, Wenjing Chen, Xinyi Han, Yuchen Li, Chunlei Zhang, Xu Pan, Tao Chen, Zhengguo Xiao, Qiyuan He, Fei Li, Zhimin Fang, Xiao Cheng Zeng, Zonglong Zhu, Shangfeng Yang

**Affiliations:** State Key Laboratory of Precision and Intelligent Chemistry, Collaborative Innovation Center of Chemistry for Energy Materials (iChEM), Anhui Laboratory of Advanced Photon Science and Technology, Department of Materials Science and Engineering, University of Science and Technology of China, Hefei 230026, China; Department of Chemistry, City University of Hong Kong, Hong Kong 999077, China; Anhui Province Key Laboratory of Structure and Functional Regulation of Hybrid Materials, Department of Chemistry, College of Chemistry and Chemical Engineering, Anhui University, Hefei 230601, China; Department of Materials Science and Engineering, City University of Hong Kong, Hong Kong 999077, China; Key Laboratory of Photovoltaic and Energy Conservation Material, Institute of Solid-State Physics (ISSP), Hefei Institutes of Physical Science (HIPS), Chinese Academy of Sciences, Hefei 230021, China; State Key Laboratory of Precision and Intelligent Chemistry, Collaborative Innovation Center of Chemistry for Energy Materials (iChEM), Anhui Laboratory of Advanced Photon Science and Technology, Department of Materials Science and Engineering, University of Science and Technology of China, Hefei 230026, China; State Key Laboratory of Precision and Intelligent Chemistry, Collaborative Innovation Center of Chemistry for Energy Materials (iChEM), Anhui Laboratory of Advanced Photon Science and Technology, Department of Materials Science and Engineering, University of Science and Technology of China, Hefei 230026, China; State Key Laboratory of Precision and Intelligent Chemistry, Collaborative Innovation Center of Chemistry for Energy Materials (iChEM), Anhui Laboratory of Advanced Photon Science and Technology, Department of Materials Science and Engineering, University of Science and Technology of China, Hefei 230026, China; Anhui Province Key Laboratory of Structure and Functional Regulation of Hybrid Materials, Department of Chemistry, College of Chemistry and Chemical Engineering, Anhui University, Hefei 230601, China; State Key Laboratory of Precision and Intelligent Chemistry, Collaborative Innovation Center of Chemistry for Energy Materials (iChEM), Anhui Laboratory of Advanced Photon Science and Technology, Department of Materials Science and Engineering, University of Science and Technology of China, Hefei 230026, China; State Key Laboratory of Precision and Intelligent Chemistry, Collaborative Innovation Center of Chemistry for Energy Materials (iChEM), Anhui Laboratory of Advanced Photon Science and Technology, Department of Materials Science and Engineering, University of Science and Technology of China, Hefei 230026, China; State Key Laboratory of Precision and Intelligent Chemistry, Collaborative Innovation Center of Chemistry for Energy Materials (iChEM), Anhui Laboratory of Advanced Photon Science and Technology, Department of Materials Science and Engineering, University of Science and Technology of China, Hefei 230026, China; Department of Chemistry, City University of Hong Kong, Hong Kong 999077, China; Key Laboratory of Photovoltaic and Energy Conservation Material, Institute of Solid-State Physics (ISSP), Hefei Institutes of Physical Science (HIPS), Chinese Academy of Sciences, Hefei 230021, China; State Key Laboratory of Precision and Intelligent Chemistry, Collaborative Innovation Center of Chemistry for Energy Materials (iChEM), Anhui Laboratory of Advanced Photon Science and Technology, Department of Materials Science and Engineering, University of Science and Technology of China, Hefei 230026, China; State Key Laboratory of Precision and Intelligent Chemistry, Collaborative Innovation Center of Chemistry for Energy Materials (iChEM), Anhui Laboratory of Advanced Photon Science and Technology, Department of Materials Science and Engineering, University of Science and Technology of China, Hefei 230026, China; Department of Materials Science and Engineering, City University of Hong Kong, Hong Kong 999077, China; Anhui Province Key Laboratory of Structure and Functional Regulation of Hybrid Materials, Department of Chemistry, College of Chemistry and Chemical Engineering, Anhui University, Hefei 230601, China; Institute of Technology for Carbon Neutralization, Yangzhou University, Yangzhou 225127, China; Department of Materials Science and Engineering, City University of Hong Kong, Hong Kong 999077, China; Department of Chemistry, City University of Hong Kong, Hong Kong 999077, China; State Key Laboratory of Precision and Intelligent Chemistry, Collaborative Innovation Center of Chemistry for Energy Materials (iChEM), Anhui Laboratory of Advanced Photon Science and Technology, Department of Materials Science and Engineering, University of Science and Technology of China, Hefei 230026, China

**Keywords:** perovskite solar cells, fullerene derivatives, ion migration, efficiency, interfacial stability

## Abstract

Solution-processible fullerene derivatives have been extensively used as electron transport layers (ETLs) of inverted perovskite solar cells (PSCs); however, the commonly used 58π-electron [6,6]-phenyl-C_61_-butyric acid methyl ester (PCBM) often tends to dimerize, especially under continuous illumination, severely compromising long-term stability. Herein, we develop a 1,4-unsymmetrical addition strategy and synthesize two novel 56π-electron fullerene derivatives bearing multiple bulky functional groups such as *tert*-butyl, indole and azaindole, designated as C_60_-TFB and C_60_-TFP, which not only play the role of anti-dimerization but also leverage a combined passivating effect of various heteroatom-containing functional groups. These groups establish robust interfacial bonding, enhancing interfacial stability by inhibiting the migration of iodide ions (I^−^) and silver (Ag). Consequently, C_60_-TFB and C_60_-TFP exhibit excellent optoelectronic properties, enabling favorable energy level alignment with perovskites. PSC devices based on C_60_-TFB and C_60_-TFP ETLs achieve a significantly enhanced power conversion efficiency (PCE) of 25.55% and 25.93%, respectively, relative to a 24.08% PCE for the PCBM-based control devices. After over 1000 h of continuous illumination at 55°C, the optimized C_60_-TFP–based devices demonstrate excellent stability, retaining 81.9% of their initial efficiency, whereas only 62.9% retention is achieved for the PCBM-based control device, indicating a dramatic enhancement of operational stability.

## Teaser

We develop novel solution-processed fullerene derivatives bearing bulky functional groups as electron transport layers for inverted perovskite solar cells, achieving a high efficiency of 25.93% along with enhanced interfacial stability.

## INTRODUCTION

Due to the outstanding optoelectronic properties of organic-inorganic hybrid perovskite materials, the power conversion efficiency (PCE) of perovskite solar cells (PSCs) has rapidly increased from 3.8% in 2009 to more than 26% [1–4]. The high efficiency, low cost, solution processibility, and flexibility make PSCs a promising technology for the next generation of advanced photovoltaics [5]. With the development of PSCs, the device structures can primarily be categorized into conventional (n-i-p) and inverted (p-i-n) configurations [6]. In comparison to conventional devices, inverted devices exhibit higher efficiency, negligible hysteresis, and greater potential for tandem applications [7–9]. Highly-efficient and stable inverted PSCs have garnered increasing interest from research institutions and enterprises around the world [6,10].

To fabricate highly efficient and stable inverted PSCs, most optimization strategies have focused on depositing high-quality perovskite films, exploring effective defect passivation methods, and developing novel charge transport materials, such as self-assembled monolayers (SAMs) [3,11–16]. By leveraging these advanced techniques, inverted devices have achieved the highest certified PCE for PSCs. Despite the wide variety of novel hole transport and passivation materials, the electron transport layers (ETLs) remain predominantly based on fullerene materials, particularly C_60_ and its derivative such as 58π-electron [6,6]-phenyl-C_61_-butyric acid methyl ester (PCBM) [2,17,18]. C_60_ is currently the most widely used ETL for high-efficiency inverted PSCs due to its ability to form a dense and uniform layer through vacuum deposition. However, its limited solubility in organic solvent generally requires vacuum deposition, thus the equipment costs significantly increase when fabricating large-area devices [19–22]. In contrast, PCBM can be deposited using a solution method, which is promising for producing large-area films at a lower cost. Nevertheless, due to its self-aggregation characteristics, solution-processed PCBM may inevitably dimerize and consequently generate pinholes in large-area films, which can severely compromise both device efficiency and stability [23,24]. When pinholes are formed, silver (Ag) atoms from the cathode and iodide (I) ions (I^−^) within the halide perovskite will interdiffuse through these pinholes, resulting in device degradation [25–29]. Although some optimized deposition techniques have been developed to deposit dense and uniform PCBM layers and have achieved decent results [30–32], the limited functional groups in PCBM are insufficient to form strong bonds with the perovskite crystal and effectively passivate surface defects, particularly regarding I^−^ ion migration. This leads to an imperfect perovskite/ETL interface, significantly limiting further improvements in interfacial stability. Therefore, it is imperative to develop novel ETLs that simultaneously improve efficiency and stability.

To design high-performance solution-processible fullerene derivative ETLs, several critical factors should be carefully considered. First, the solubility of fullerene derivatives in non-polar solvents should be maximized to ensure its full coverage on the perovskite films. Second, fullerene derivatives must possess appropriate energy levels and high carrier mobility to facilitate efficient electron extraction and transport. Third, they can maintain stable morphology to prevent perovskite layers from degradation induced by moisture and oxygen in the environment, as well as metal migration from the electrodes. Last but not least, it would be more advantageous if the fullerene derivative could demonstrate strong defect passivation capabilities to minimize non-radiative recombination [23]. In particular, compared to widely reported 58π-electron C_60_ derivatives, 56π-electron C_60_ derivatives exhibit a more compatible energy level structure as ETL materials for perovskite solar cells [33,34]. Additionally, their larger number of modification sites allows for the introduction of more bulky functional groups, and their unique tetra-functionalized structure is also expected to be capable of inhibiting dimerization. For instance, Li *et al.* synthesized an indene-C_60_ bisadduct ICBA, which was subsequently utilized to fabricate highly efficient PSCs [33]. Wei *et al.* precisely synthesized the fullerene bisadduct C_60_BB, which features a higher-lying conduction band minimum, thereby enhancing high performance of tin-based PSCs [34]. Matsuo *et al.* synthesized an evaporable fullerene bisadduct *t*BU-FIDO, notably improving device stability [35]. It is noteworthy that fullerene derivatives capable of inhibiting dimerization have been scarcely reported. Hence, design and synthesis of novel anti-dimerization fullerene ETLs is highly desirable.

Herein, we developed a 1,4-unsymmetrical addition strategy and synthesized two novel 56π-electron fullerene derivatives bearing multiple bulky functional groups such as *tert*-butyl and azaindole, designated as C_60_-TFB and C_60_-TFP, which exhibit outstanding anti-dimerization ability and contain various heteroatom-containing functional groups. These functional groups are capable of passivating surface defects of perovskite film and can also form robust interfacial bonds, enhancing interfacial stability by inhibiting the migration of I^−^ ions on the perovskite surface. Furthermore, these materials demonstrate excellent optoelectronic properties with favorable energy level alignment with perovskites. Consequently, PSC devices based on C_60_-TFB and C_60_-TFP ETLs achieved significantly improved efficiencies of 25.55% and 25.93%, respectively, relative to a 24.08% efficiency for the control devices based on PCBM ETL. Additionally, after >1000 h of continuous illumination at 55°C, the optimized C_60_-TFP–based devices demonstrated remarkably enhanced operational stability, retaining 81.9% of their initial efficiency.

## RESULTS AND DISCUSSION

### Molecular design, synthesis, and characterization

In comparison to the conventional 1,2-addition fullerene derivatives, the 1,4-unsymmetrical adducts of C_60_ exhibit a higher lowest unoccupied molecular orbital (LUMO) energy level and enhanced visible light absorption [36–38]. In addition, their solubility, film-forming properties, and electrical properties can be optimized by varying the combinations of 1,4-bifunctionalization [39,40]. Although 1,4-unsymmetrical C_60_ bisadducts have significant potential for application in PSCs, to the best of our knowledge, due to the synthetic challenge, there have been no reports on 56π-fullerene derivative ETLs based on 1,4-unsymmetrical bisadducts of C_60_ to date. Previous studies have shown that specific heteroatoms and functional groups containing heteroatoms in fullerene derivatives, such as fluorine atoms [41,42], pyridine [43–46], and ester groups [34,47,48], play a key role in improving the performance of fullerene derivative ETLs and enhancing the stability of PSC devices. Moreover, the presence of the bulky *tert*-butyl (*t*Bu) group has been demonstrated to significantly influence the self-assembly behavior of fullerene molecules due to its considerable steric hindrance, which is a crucial factor in regulating fullerene aggregation [35]. Our molecular design strategy aims to leverage the synergistic effects of these functional groups in order to enhance the performance of photovoltaic devices. Accordingly, we propose the possibility of constructing a new class of tetra-functionalized 56π-electron fullerene derivative ETL, which include various heteroatom-containing functional groups and bulky *tert*-butyl groups, through a stepwise approach involving 1,4-unsymmetrical bifunctionalization and site-selective cycloaddition of C_60_. It should be noted that multi-functionalized multiadducts of C_60_ are very difficult to synthesize with isomeric purity. To the best of our knowledge, C_60_-TFB and C_60_-TFP possess a unique tetra-functionalized 56π-electron structure, which includes both 1,4-asymmetrical bisaddition and [3 + 2] cycloaddition, and cannot be easily synthesized by using any other reported methods.

**Figure 1.**
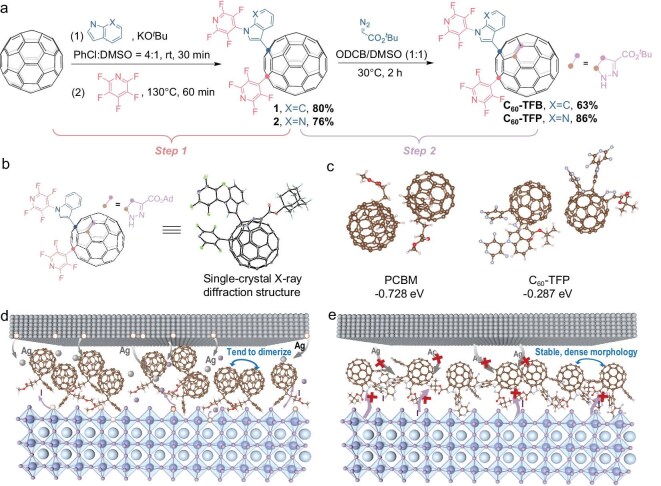
(a) Synthetic route for the novel 56π-electron C_60_ derivatives C_60_-TFB and C_60_-TFP. Step 1: one-pot synthesis of a 1,4-unsymmetrical skeleton of C_60_ while incorporating fluorine atoms and pyridine groups. Step 2: site-selectively constructing a tetra-functionalized 56π-electron C_60_ skeleton and further introducing tert-butyl, ester, and 1H-pyrazole groups. (b) Single-crystal X-ray diffraction structure of an analogous compound with adamantine substituting the tert-butyl group of C_60_-TFB. (c) Intermolecular interactions of PCBM and C_60_-TFP dimers predicted by DFT calculations. (d, e) Schematic diagrams of (d) PCBM (easy-dimerization) and (e) C_60_-TFP films (anti-dimerization) on the perovskite layer.

The synthetic route for the novel 56π-electron fullerene derivatives is designed as follows (Fig. 1a): in the first step, a one-pot 1,4-unsymmetrical bifunctionalization of C_60_ with indole/7-azaindole and pentafluoropyridine is achieved by using KO*^t^*Bu as a promoter, resulting in the formation of a bifunctionalized C_60_ framework containing fluorine and pyridine groups [49]. In the second step, the 1,4-unsymmetrical bisadducts serve as precursors for a site-selective [3 + 2] cycloaddition reaction with *tert*-butyl diazoacetate, introducing *tert*-butyl and ester groups [50]. This step also facilitates the incorporation of a nitrogen-containing 1H-pyrazoline ring on C_60_, ultimately yielding isomer-pure novel 56π-electron fullerene derivatives, designated as C_60_-TFB and C_60_-TFP. Notably, the two-step method for selectively synthesizing tetra-functionalized 56π-electron C_60_ derivatives, C_60_-TFB and C_60_-TFP, is highly efficient, with each step yielding >60%. The overall yield based on the consumption of the starting material C_60_ reaches 50% and 65%, respectively [50]. This demonstrates that this new type of 56π-electron fullerene ETL offers advantages in terms of efficient and convenient synthesis at a low cost.

The molecular structures of C_60_-TFB and C_60_-TFP were characterized by using ^1^H, ^1^F, and ^13^C Nuclear Magnetic Resonance (NMR) spectroscopy (Figs S1–S12), as well as matrix-assisted laser desorption/ionization time-of-flight (MALDI-TOF) mass spectrometry (Fig. S13). The molecular structure of an analogous compound with adamantine substituting the *tert*-butyl group was unambiguously determined by single-crystal X-ray diffraction (Fig. 1b), rationally confirming the proposed chemical structures of C_60_-TFB and C_60_-TFP (see Note S14 in Supporting Information for details) [50].

The energy levels of PCBM, C_60_-TFB, and C_60_-TFP were determined by using cyclic voltammetry in conjunction with UV-vis absorption spectroscopy (Figs S14 and S15). Notably, the 56π single-isomer C_60_ multiadducts, modified through 1,4-unsymmetrical addition and 1,2-cycloaddition, incorporate a *tert*-butyl group that introduces an exceptionally large steric hindrance. This pronounced steric effect is considered to be a critical factor in modulating the assembly behavior of fullerene molecules and effectively inhibiting their aggregation [35,51], allowing them to maintain a stable and dense morphology without pinholes, even after prolonged device operation. To verify the steric effect of our newly synthesized molecules on their dimerization, we first investigated the interactions of carbon cages in PCBM and C_60_-TFP using density functional theory (DFT) to assess the possibility of aggregation behavior. As shown in Fig. 1c, the binding energy for C_60_-TFP molecules is −0.287 eV, which is more negative than that of PCBM molecules (−0.728 eV). This indicates a weaker intermolecular interaction for the C_60_-TFP molecules compared to the PCBM, which is likely due to the steric-hindrance effect. Besides, the improved film property of ETL is expected to inhibit dimerization of fullerene molecules and further prevent the interdiffusion of the Ag atoms from the cathode and I ions from the perovskite (Fig. 1d, e), as discussed in detail below.

### Photostability of the new C_60_ derivative ETLs

**Figure 2.**
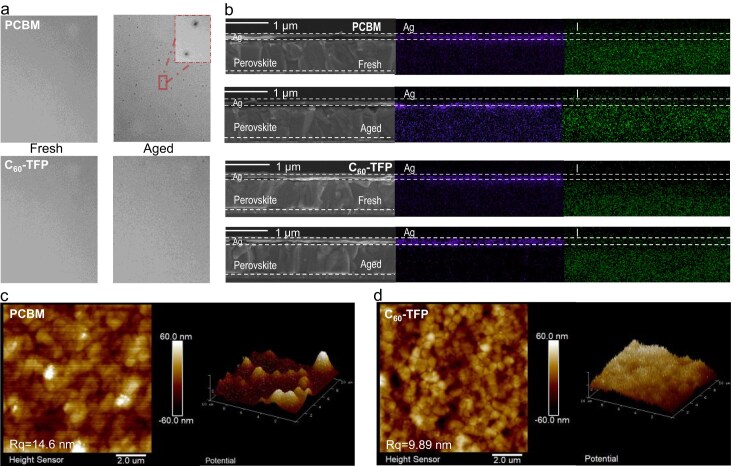
(a) Optical microscopy photographs of Ag electrodes of devices with PCBM/C_60_-TFB, in their initial state and after continuous simulated AM1.5 G illumination aging for 800 h. (b) Cross-sectional SEM images of the devices in their fresh state and after 1000 h of continuous simulated AM1.5 G illumination aging along with EDX mapping of Ag and I. (c, d) AFM images and the potential images from KPFM of the (c) PCBM and (d) C_60_-TFP on the perovskite substrates after continuous simulated AM1.5 G illumination aging for 800 h.

During the operation of PSC devices, the intrinsic characteristics of perovskite materials make them susceptible to ion and metal migrations [25,52,53]. Specifically, iodide ions (I^−^) tend to diffuse toward the Ag electrode, leading to corrosion, while the simultaneous migration of Ag into the perovskite layer significantly compromises long-term operational durability. To evaluate the stability of devices incorporating novel C_60_ derivative ETLs during operation, optical microscopy was employed to examine the surface morphology of Ag electrodes both before and after 800-h aging. Aged devices based on PCBM exhibited large black voids, which can be attributed to the reactions between Ag and I^−^, resulting in the formation of AgI [25,54,55]. In contrast, devices utilizing C_60_-TFB and C_60_-TFP ETLs demonstrated relatively stable surface morphologies of the Ag electrodes after aging (Fig. 2a, Fig. S16a).

To gain further insight into the internal changes occurring within the devices, cross-sectional SEM images were acquired (Fig. 2b, Fig. S16c). Significant migration of Ag into the perovskite layer, accompanied by uneven cross-sectional morphology, can be seen after 1000-h aging. Simultaneously, the perovskite layer experiences notable degradation, characterized by the diffusion of iodide ions toward the Ag electrode. This degradation is most likely driven by the aggregation of PCBM. In contrast, devices incorporating the novel ETLs demonstrated well-maintained electrodes and only minimal structural changes in the perovskite layer, while the migration of Ag and iodide ions was significantly inhibited. These observations underscore the crucial role of novel fullerene derivatives in mitigating the migration of Ag and iodide ions. By protecting the Ag electrode and the perovskite layer from deterioration, these derivatives are expected to considerably improve the long-term operational stability of PSCs as discussed below.

Furthermore, atomic force microscopy (AFM) was used to capture images of PCBM, C_60_-TFB, and C_60_-TFP after 800 h of illumination (Fig. 2c and d; Figs S16b and S17). Devices incorporating C_60_-TFB and C_60_-TFP exhibited negligible changes on both the morphology and root mean square roughness (RMS) value, while PCBM-based devices displayed prominent bright protrusions, indicative of dimerization and the formation of larger aggregates [26,56]. Such aggregation impaired the ability of PCBM to uniformly and densely cover the perovskite layer, resulting in direct interactions between the perovskite and the Ag electrode, which severely degraded the operational reliability of PSCs.

### Interactions between new C_60_ derivative ETLs and perovskite

**Figure 3.**
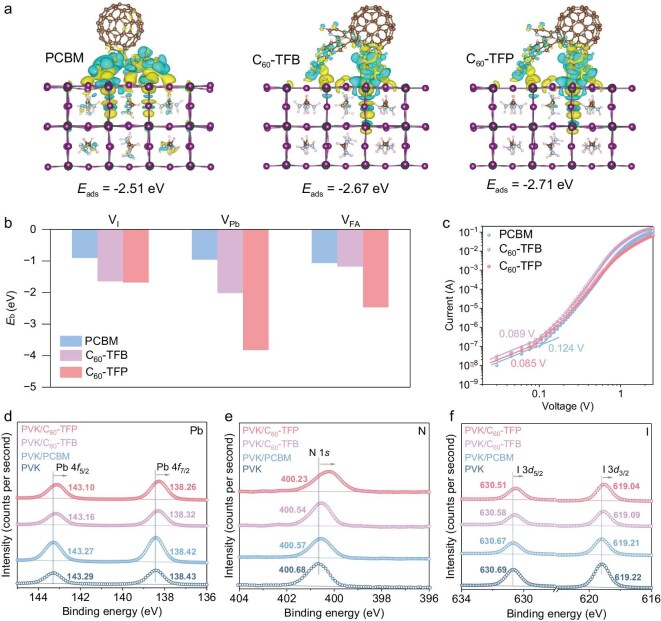
(a) Theoretically calculated adsorption energy (*E_ads_*) between the perovskite layer and PCBM, C_60_-TFB, and C_60_-TFP molecules. (b) Absorption energy (*E_b_*) of PCBM, C_60_-TFB, and C_60_-TFP on different types of defects within perovskite. (c) Dark current-voltage responses of the electron-only FTO/SnO_2_/perovskite/ETL/Ag devices. XPS spectra of Pb 4f (d), N 1s (e) and I 3d (f) detected for the pristine perovskite (PVK), perovskite/PCBM, perovskite/C_60_-TFB, and perovskite/C_60_-TFP films.

Beyond the intrinsic structural stability of the new fullerene ETLs, which supports the long-term operational stability of the device, the interfacial interactions between the ETL and the perovskite layer are crucial for facilitating defect passivation and further strengthening interfacial stability [57–59]. To gain a deeper understanding of these interactions, density functional theory (DFT) calculations were performed to evaluate the interactions between PCBM, C_60_-TFB, and C_60_-TFP with the perovskite layer. The orientations of the ETLs were first identified (Fig. S18), followed by the calculation of interfacial binding energies [26]. As shown in Fig. 3a, the adsorption energy (*E_ads_*) between PCBM and the perovskite layer was −2.51 eV, while C_60_-TFB and C_60_-TFP exhibited higher adsorption energies of −2.67 eV and −2.71 eV, respectively. A more negative *E_ads_* value indicates stronger coordination interactions of the ETL with the perovskite, which aids in passivating perovskite surface defects [9]. Moreover, C_60_-TFB and C_60_-TFP enhanced the coordination around I, Pb, and FA vacancies, effectively mitigating the impact of surface vacancy defects and achieving a robust passivation effect (Fig. 3b; Figs S19–S21).

To further assess the defect density of perovskite films coated with PCBM, C_60_-TFB, and C_60_-TFP, electron-only devices with an ITO/SnO_2_/perovskite/ETL/Ag structure were fabricated, and the corresponding I-V curves were measured (Fig. 3c). The trap-filled limit voltages for PCBM, C_60_-TFB, and C_60_-TFP were determined to be 0.124 V, 0.089 V, and 0.085 V, respectively. Based on these measurements, the calculated defect densities for PCBM, C_60_-TFB, and C_60_-TFP were 1.33 × 10^15^ cm^−3^, 9.58 × 10^14^ cm^−3^, and 9.15 × 10^14^ cm^−3^, respectively. These findings demonstrate that C_60_-TFB and C_60_-TFP can effectively passivate surface defects in perovskite films, significantly reducing defect density.

To gain a deeper understanding of the interactions between the ETL and the perovskite surface, X-ray photoelectron spectroscopy (XPS) measurements were conducted. As shown in Fig. 3d, the pristine perovskite film exhibited binding energies for Pb 4f_5/2_ and Pb 4f_7/2_ at 143.29 eV and 138.43 eV, respectively. While PCBM deposition led to negligible signal shifts, after C_60_-TFB and C_60_-TFP deposition, these binding energies made an obvious shift to lower values, primarily due to the donation of lone-pair electrons from the oxygen atoms in the −COO− groups of the ETLs to the vacant 6p orbitals of Pb^2+^ ions. This electron donation increased the electron density around Pb^2+^, resulting in a negative shift in the binding energies of Pb 4f_5/2_ and Pb 4f_7/2_. Likewise, the binding energies of N 1s and I 3d within perovskite shifted negatively as well, further confirming the strong interfacial interactions between the perovskite and the ETL (Fig. 3e and f) [44,60].

Fourier-transform infrared (FTIR) spectroscopy was employed to further investigate the interactions between the ETL and the perovskite. As seen in Fig. S22, shifts in the stretching vibrations of −COO−, 2,3,5,6-tetrafluoropyridine, and −C-F− were observed, confirming robust interactions between the ETL and the perovskite [61].

### Carrier transport dynamics between the new C_60_ derivative ETLs and perovskite

**Figure 4.**
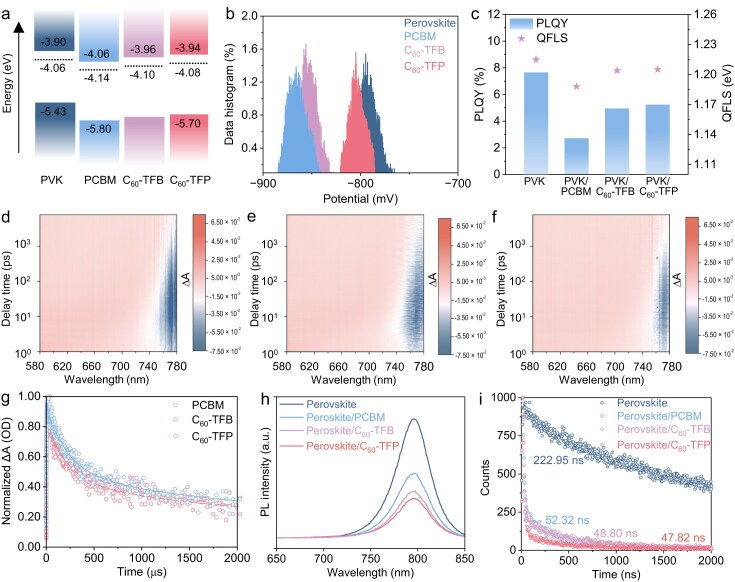
(a) Energy-level diagram for the perovskite film, PCBM, C_60_-TFB, and C_60_-TFP. (b) Statistical distribution of surface potential extracted from the KPFM images. (c) The PLQY and corresponding QFLS of perovskite films before and after depositing PCBM, C_60_-TFB, and C_60_-TFP. The substrate for these samples is FTO. (d–f) The pseudocolor plots of glass/perovskite/ETL films excited at 400 nm. (g) Normalized decay kinetic curves at 775 nm of the TA spectra. (h) The PL spectra of perovskite/PCBM, perovskite/C_60_-TFB, and perovskite/C_60_-TFP deposited on glass. (i) TRPL spectra of perovskite/PCBM, perovskite/C_60_-TFB, and perovskite/C_60_-TFP deposited on glass.

To investigate carrier transport at the interface, ultraviolet photoelectron spectroscopy (UPS) and UV-visible absorption measurements were conducted to assess the energy level structures of the perovskite films, PCBM, C_60_-TFB, and C_60_-TFP (Fig. 4a, Fig. S23). It was observed that the Fermi levels of both C_60_-TFB and C_60_-TFP were elevated. Furthermore, Kelvin probe force microscopy (KPFM) analysis revealed that the work function of C_60_-TFP is much closer to that of the perovskite surface (Fig. 4b, Fig. S24). Collectively, these results suggest that the energy band alignment between C_60_-TFP and the perovskite layer is more favorable for electron transport, which reduces carrier accumulation at the interface and significantly inhibits charge recombination [25,26]. To quantitatively evaluate non-radiative recombination, photoluminescence quantum yield (PLQY) measurements were performed on perovskite, perovskite/PCBM, perovskite/C_60_-TFB, and perovskite/C_60_-TFP films. As illustrated in Fig. 4c, the PLQY of the perovskite film decreased from 7.65% to 2.68% following the application of PCBM. In contrast, the reduction in PLQY for the perovskite/C_60_-TFB and perovskite/C_60_-TFP films was significantly lower. Additionally, the quasi-Fermi level splitting (QFLS) was calculated using the following equation:


(1) \begin{eqnarray*}
\textit{QFLS} = {\kappa }_BT*\ln\! \left( {\textit{PLQY} \times {J}_G/{J}_{0,rad}} \right),
\end{eqnarray*}

where *J_G_* represents the generated current density under 1 sun illumination, *J*_0_,*_rad_* denotes the radiative recombination current in dark condition [62]. After the perovskite surface was coated with PCBM, the perovskite/PCBM film exhibited a QFLS value of 1.188 V. In contrast, the perovskite/C_60_-TFB and perovskite/C_60_-TFP films displayed QFLS values of 1.204 V and 1.205 V, respectively, which are remarkably close to that of pristine perovskite. This suggests that the interfacial recombination losses of perovskite/ETL have been effectively mitigated. These results indicate that the smaller energy level offset between the new ETLs and the perovskite effectively suppresses non-radiative recombination at the interface. To gain deeper insight into charge recombination dynamics, ultrafast transient absorption (TA) characterization was subsequently employed. The pseudocolor plots of the glass/perovskite/ETL films excited at 400 nm (Fig. 4d and f), along with the decay kinetic curves at 775 nm extracted from ultrafast transient absorption spectroscopy (TAS) measurements, demonstrate that the decay time (τ_1_) for photoexcited electron injection from the perovskite to the ETL decreases from 30.15 ps for the PCBM to 15.99 ps for the C_60_-TFP (Fig. 4g, Fig. S25, Table S2). This indicates facilitated charge transfer at the perovskite/C_60_-TFP interface. Furthermore, steady-state photoluminescence (PL) and time-resolved photoluminescence (TRPL) measurements were conducted to study charge extraction and recombination at the perovskite/ETL interface. As shown in Fig. 4h, the PL intensity of perovskite films covered with ETLs exhibits significant quenching compared to the pristine perovskite film, indicating effective electron extraction between the perovskite and ETLs. Additionally, C_60_-TFP results in a much lower PL intensity than PCBM, suggesting a faster electron extraction capability. Figure 4i displays the TRPL spectra of the perovskite film and perovskite films covered with different ETLs. From the data fitted with a bi-exponential function, the carrier lifetimes are 52.33 ns, 48.80 ns, and 47.82 ns for perovskite/PCBM, perovskite/C_60_-TFB, and perovskite/C_60_-TFP, respectively (Table S3). This is consistent with the results of the TA measurement, which further confirms the efficient electron extraction at the perovskite/C_60_-TFP interface.

### Device performance and stability

**Figure 5.**
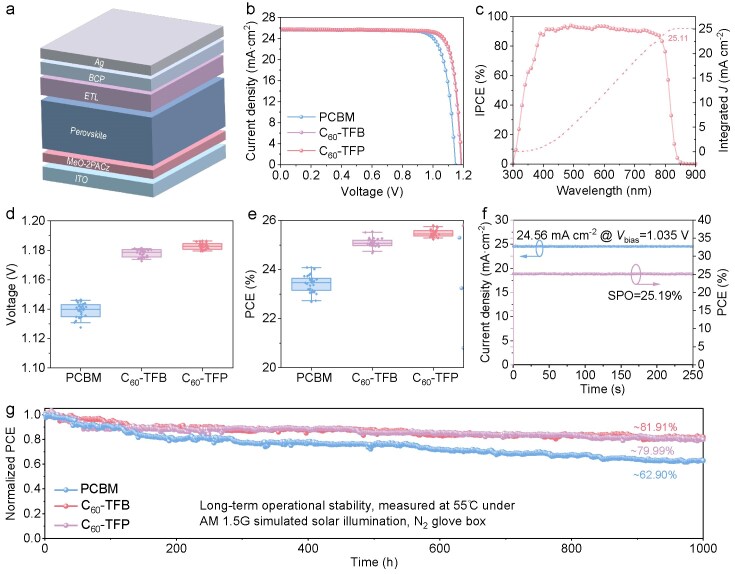
(a) Device structure of inverted PSCs. (b) *J–V* curves of the best-performing PSC devices with PCBM, C_60_-TFB, and C_60_-TFP ETLs, respectively. (c) EQE spectrum of the best-performing C_60_-TFP devices. (d, e) Statistics of *V*_OC_ (d) and PCE (e) for PCBM, C_60_-TFB, and C_60_-TFP devices. (f) The stabilized photocurrent and PCE output of the C_60_-TFP device were measured at the MPP. (g) The evolution of the normalized PCE of the unencapsulated device under the LED illumination (100 mW cm^−2^) at 55°C in a N_2_ glovebox.

To evaluate the effect of fullerene ETL on device performance, devices with a configuration of ITO/MeO-2PACz/perovskite/ETL/BCP/Ag were fabricated, in which MeO-2PACz and BCP are [2-(3,6-dimethoxy9H-carbazol-9-yl)ethyl]phosphonic acid and bathocuproine, respectively (Fig. 5a). Figure 5b displays the current-voltage (*J-V*) curves of the best-performing devices with different ETLs, and their photovoltaic parameters are summarized in Table 1. The PCBM-based best-performing device demonstrates a PCE of 24.08%, with an open-circuit voltage (*V*_OC_) of 1.146 V, a short-circuit density (*J*_SC_) of 25.64 mA cm^−2^, and a fill factor (FF) of 81.92%. In comparison, the C_60_-TFB–based best device exhibits an improved PCE of 25.55%, with a *V*_OC_ of 1.181 V, a *J*_SC_ of 25.71 mA cm^−2^, and an FF of 84.15%. The C_60_-TFP–based best device achieved the highest PCE of 25.93% with a *V*_OC_ of 1.185 V, a *J*_SC_ of 25.77 mA cm^−2^, and FF of 84.92%. The external quantum efficiency (EQE) for the champion target device, shown in Fig. 5c, indicates an integrated *J*_SC_ of 25.11 mA cm^−2^, which is close to the value obtained from the *J-V* curves (25.76 mA cm^−2^, Table 1).

**Table 1.** Best photovoltaic parameters of PSCs with various ETLs.

ETL	*V*_OC_ (V)	*J*_SC_ (mA/cm^2^)	FF (%)	PCE (%)
PCBM	1.146	25.64	81.92	24.08
C_60_-TFB	1.181	25.71	84.15	25.55
C_60_-TFP	1.185	25.77	84.92	25.93

To assess the reproducibility of device performance with different ETLs, 30 control and target devices were independently fabricated simultaneously under the same conditions. The statistical photovoltaic parameters are presented in Fig. 5d, e, and Fig. S26, demonstrating improved reproducibility of the target devices. Analysis of the statistical photovoltaic parameters reveals that the enhanced PCE of the target device is primarily attributed to the improvements of *V*_OC_ and FF. The C_60_-TFB–based device achieved a stabilized power output (SPO) of 25.19% at its maximum power point (*V*_max_ = 1.035 V) after 250 s of continuous 1 sun illumination (Fig. 5f). Whether the intrinsic stability of the new C_60_-TFB and C_60_-TFP ETLs can improve device photostability is first assessed by measuring the long-term operational stability of the PSC devices (Fig. 5g). After 1000 h of continuous illumination at 55°C in a N_2_ glovebox, the unencapsulated devices based on C_60_-TFB and C_60_-TFP ETLs demonstrated retention rates of 80.0% and 81.9% of their initial efficiency, respectively. In contrast, the PCBM-based device retained only 62.9%. This significantly enhanced operational durability can be primarily attributed to the superior interfacial stability arising from the novel fullerenes and their high intrinsic stability. The effects of ETLs on the stability, specifically humidity and thermal stability, of perovskite films and devices were also investigated. The water contact angles of the PCBM, C_60_-TFB, and C_60_-TFP films were measured to be 80.2°, 96.5°, and 96.6°, respectively (see Fig. S5g inset), indicating improved moisture resistance for the perovskite films. This could be attributed to the hydrophobicity of fluorine atoms within the C_60_-TFB and C_60_-TFP molecules. Indeed, the humidity stability of unencapsulated devices was monitored under ambient conditions (temperature: 20 ± 5°C, relative humidity: 30%). As shown in Fig. S27a, the target devices demonstrated superior humidity stability, retaining ∼86.0% and 85.0% of their initial efficiency after >1200 h of exposure, while the PCBM-based device exhibited only 67.3% efficiency retention under the same conditions. Additionally, thermal stability was evaluated by storing unencapsulated devices at 85°C in a nitrogen glovebox. Figure S27b demonstrates that the target devices exhibited significantly improved thermal stability compared to the PCBM-based device, maintaining over 81.20% and 80.00% of their original PCE after 1200 h of heating at 85°C, while the PCE of the control device decreased to 65.20% after 900 h of aging. The enhanced thermal stability is attributed to the reduction in trap states resulting from perovskite surface defect passivation induced by C_60_-TFB and C_60_-TFP, as well as stronger interfacial bonding. This interaction acts as a protective barrier, inhibiting the volatilization of organic cations and thereby stabilizing the perovskite structure [48,63]. The structures of C_60_-TFB and C_60_-TFP are very similar, with the only difference being that the indole ring in C_60_-TFP contains an additional nitrogen atom compared to that in C_60_-TFB. Therefore, the higher PCE of PSC devices based on C_60_-TFP ETL compared to those based on C_60_-TFB ETL can be attributed to this specific nitrogen atom, which provides an additional passivation effect.

## CONCLUSION

In conclusion, we developed a 1,4-unsymmetrical addition strategy and synthesized two novel 56π-electron fullerene derivatives, C_60_-TFB and C_60_-TFP bearing multiple bulky functional groups containing heteroatoms, to serve as new ETLs for inverted PSCs. Compared to PCBM, C_60_-TFB and C_60_-TFP demonstrate superior anti-dimerization properties when deposited through a solution process, due to their highly intrinsic stability, which significantly improves the uniformity of the films. Meanwhile, the heteroatom-containing functional groups in C_60_-TFB and C_60_-TFP facilitated robust interfacial bonding, which is crucial for enhancing interfacial stability by inhibiting the migration of iodide ions and Ag, thereby preventing undesirable reactions between the perovskite layer and Ag electrode. Furthermore, C_60_-TFB and C_60_-TFP achieved more favorable energy level alignment with the perovskite layer, contributing to more efficient electron extraction. As a result, devices based on C_60_-TFB and C_60_-TFP achieved impressive PCEs of 25.55% and 25.93%, respectively, and retained 81.91% and 79.99% of their initial efficiencies after >1200 h of continuous light exposure at 55°C. This study underscores the critical importance of novel 56π-electron fullerene derivative ETLs and strengthening interfacial bonding to enhance device stability. These advancements pave the way for future developments of charge transport materials toward highly stable perovskite solar cells.

## METHODS

Details of the materials and methods are available in the online supplementary file.

## Supplementary Material

nwaf466_Supplemental_File
