# Supplementary material for: Anti-dimerization 56π-electron fullerene adduct bearing bulky functional groups for inverted perovskite solar cells with enhanced interfacial stability
Source: Natl Sci Rev. 2025 Oct 30;12(12):nwaf466. doi: 10.1093/nsr/nwaf466 (PMC12696875; doi:10.1093/nsr/nwaf466)
Supplement: nwaf466_Supplemental_File [file nwaf466_supplemental_file.pdf]

Supporting Information

Anti-Dimerization 56π-Electron Fullerene Adduct Bearing Bulky Functional Groups for Inverted Perovskite Solar Cells with Enhanced Interfacial Stability

Xue Wang[1,3,†], Shenghu Yuan[2,†], Shuaihua Lu[4,†], Zheng Liang[5], Shantao Zhang[1], Rongyao Lv [1], Xinyu Li[1], Hongchang Fan[2], Wenjing Chen[1], Xinyi Han[1], Yuchen Li[1], Chunlei Zhang[3], Xu Pan[5], Tao Chen[1], Zhengguo Xiao[1], Qiyuan He[4], Fei Li[2,*], Zhimin Fang[6,*], Xiao Cheng Zeng[4,*], Zonglong Zhu[3,*] and Shangfeng Yang[1,*]

[1] State Key Laboratory of Precision and Intelligent Chemistry, Collaborative Innovation Center of Chemistry for Energy Materials (iChEM), Anhui Laboratory of Advanced Photon Science and Technology, Department of Materials Science and Engineering, University of Science and Technology of China, Hefei 230026, China

[2] Anhui Province Key Laboratory of Structure and Functional Regulation of Hybrid Materials, Department of Chemistry, College of Chemistry and Chemical Engineering, Anhui University, Hefei 230601, China

[3] Department of Chemistry, City University of Hong Kong, Kowloon 999077, Hong Kong

[4] Department of Materials Science and Engineering, City University of Hong Kong, Kowloon 999077, Hong Kong

[5] Key Laboratory of Photovoltaic and Energy Conservation Material, Institute of Solid-State Physics (ISSP), Hefei Institutes of Physical Science (HIPS), Chinese Academy of Sciences, Hefei 230021, China

[6] Institute of Technology for Carbon Neutralization, Yangzhou University, Yangzhou 225127, China

*Corresponding authors. E-mails: lfe@ahu.edu.cn; fangzm@yzu.edu.cn; zonglzhu@cityu.edu.hk; xzeng26@cityu.edu.hk; sfyang@ustc.edu.cn
[†] Equally contributed to this work.

S1. Experimental Section

S1.1 Materials

All the commercial materials were used as received. Dimethyl sulfoxide (99.7%), N, N-dimethylformamide (99.99%), chlorobenzene (99.9%), and isopropanol (99.5%) were purchased from Energy Chemical. Formamidinium iodide (FAI), lead iodide (PbI_2, 99.9985%), and [2-(3,6-Dimethoxy9H-carbazol-9-yl)ethyl]phosphonic acid (MeO-2PACz) were purchased from TCI (Japan). The cesium iodide (CsI), methylammonium iodide (MAI), C_{60}, BCP (99.9%), methylammonium chloride (MACl), and PEAI were purchased from Xi'an Polymer Light Technology Corp. (China). None of the materials have been further purified.

S1.2 Synthesis

Tert-butyl diazoacetate and solvents were obtained from commercial suppliers without further purification. 1,4-Fullerene derivatives **1** were prepared according to our previous work.[1] All reactions were carried out under an argon or nitrogen atmosphere using standard Schlenk techniques. ^{1}H NMR, ^{19}F NMR, and proton-decoupled ^{13}C NMR spectra were recorded on a 400 MHz (400 MHz for ^{1}H NMR, 376 MHz for ^{19}F NMR, 100 MHz for ^{13}C NMR) NMR spectrometer. The ^{1}H NMR spectra were referenced to 2.50 ppm residual dimethyl sulfoxide and 2.05 ppm residual acetone, and the ^{13}C NMR spectra were referenced to 39.52 ppm residual dimethyl sulfoxide and 29.85 ppm residual acetone. Column chromatography was carried out employing silica gel G.

S1.2.1 Synthesis of 2

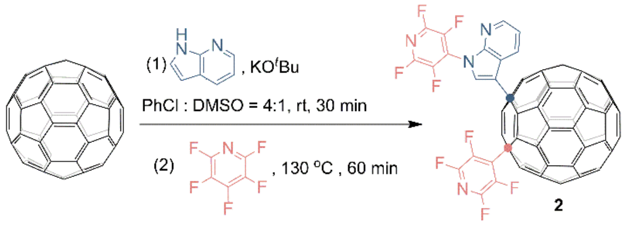

The reaction was carried out using C_{60} (0.05 mmol)/7-azaindoie (0.06 mmol, 1.2 equiv)/KO^{t}Bu (0.125mmol, 2.5 equiv) in Schlenk tubes under a N_2 atmosphere for 30 minutes. Subsequently, pentafluoropyridine (0.5 mmol, 10 equiv) was added, and the mixture was placed in a 130 °C oil bath and stirred for 60 minutes. Then, the mixture was cooled to room temperature and then evaporated in vacuo. The residue was separated on a silica gel column with carbon disulfide/dichloromethane (2:1) as the eluent to give **2** (36.3 mg, 76%) as a black amorphous solid.

^{1}**H NMR** (400 MHz, CS_2/d_6-acetone) δ 9.46 (d, J = 8.8 Hz, 1H), 9.09 (d, J = 4.8 Hz, 1H), 8.71 (s, 1H), 7.99 (m, 1H); ^{19}**F NMR** (376 MHz, CS_2/d_6-acetone) δ -87.38 to -87.71 (m, 4F), -134.34 to -134.52 (m, 4F); ^{13}**C NMR** (100 MHz, CS_2/d_6-acetone) δ 151.16, 149.47, 149.18, 149.00, 148.42, 147.93, 147.89, 147.74, 147.61, 147.52, 147.48, 147.45, 147.40, 147.23, 147.05, 146.83, 146.26, 146.21, 146.17, 146.06, 145.93, 145.26, 145.06, 145.02, 144.90, 144.86, 144.81, 144.68, 144.56, 144.54, 144.49, 144.45, 144.31, 144.14, 144.05, 144.03, 144.02, 143.85, 143.81, 143.75, 143.63, 143.52, 143.47, 143.23, 143.13, 143.00, 142.79, 142.73, 142.64, 142.32, 141.95, 141.07, 139.94, 139.69, 138.67, 138.26, 129.73, 125.77, 119.60, 119.47, 119.09, 56.04 (sp^3-C of C_{60}), 32.52 (sp^3-C of C_{60}). HRMS (MALDI-TOF-MS, DCTB as matrix, negative mode): m/z [M]- calcd for $C_{77}H_4F_8N_4$ 1136.0308; found 1136.0312.

S1.2.2 Synthesis of C_{60}-TFB

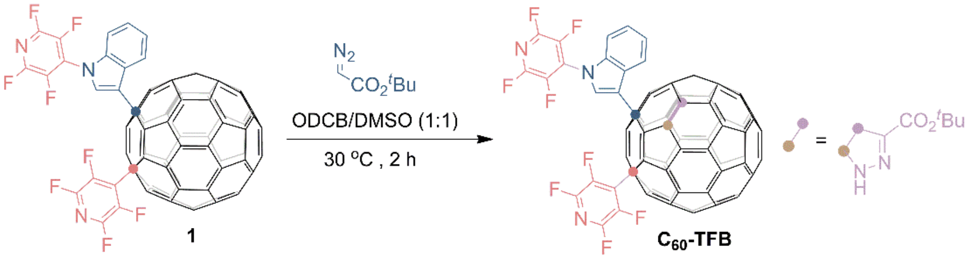

1,4-Fulleroindole **1** (29.4 mg, 0.025 mmol) and tert-butyl diazoacetate (18.0 mg, 0.125 mmol) were dissolved in ODCB (5 mL) and DMSO (5 mL) at room temperature under N_2 atmosphere. After full dissolution, the reaction solution was stirred in an oil bath at 30 °C for 2 h. Then, the mixture was cooled to room temperature and then evaporated in vacuo. The residue was separated on a silica gel column with carbon disulfide/dichloromethane/ethyl acetate (12:4:0.5) as the eluent to give **C_{60}TFB** (20.8 mg, 63%) as black amorphous solid. ^{1}**H NMR** (400 MHz, CS_2/d_6-acetone) δ 9.27 (s, 1H), 8.25 (d, J = 8.0 Hz, 1H), 8.10 (s, 1H),7.38 (d, J = 4.8 Hz, 2H), 7.27-7.23 (m, 1H),1.41 (s, 9H); ^{19}**F NMR** (376 MHz, CS_2/d_6-acetone) δ -88.99 to -89.16 (m, 4F), -133.90 to -135.06 (m, 4F); ^{13}**C NMR** (100 MHz, CS_2/d_6-acetone) δ 160.02 (ester C), 152.56, 151.98, 150.60, 150.60, 150.02, 150.01, 149.75, 148.66, 148.51, 148.42, 148.29 148.10, 147.59, 147.51, 147.37, 147.28, 147.26, 146.96, 146.91, 146.59, 146.58, 146.55, 146.46, 146.23, 146.02, 145.96, 145.94, 145.91, 145.80, 145.58, 145.49, 145.31, 145.15, 145.11, 144.90, 144.85, 144.32, 144.21, 144.05, 143.92, 143.39, 142.88, 142.57, 142.11, 142.08, 141.24, 141.11, 140.86, 138.61, 137.32, 137.12, 135.15, 127.39 (aryl C),

126.69 (aryl C), 125.19 (aryl C), 123.13 (aryl C), 122.08 (aryl C), 117.82 (aryl C), 112.58 (aryl C), 112.55 (aryl C), 112.53 (aryl C), 87.88 (sp^3-C of C_{60}), 81.87 (sp^3-C of C_{60}), 71.49 (sp^3-C of C_{60}), 55.65 (sp^3-C of C_{60}), 28.47 (-CH_3). UV-vis ($CHCl_3$) λ_{max} 353, 452 nm; HRMS (MALDI-TOF-MS, DCTB as matrix, negative mode): m/z [M]- calcd for $C_{84}H_{15}F_8N_5O_2$ 1278.06; found 1278.08.

S1.2.3 Synthesis of C_{60}-TFP

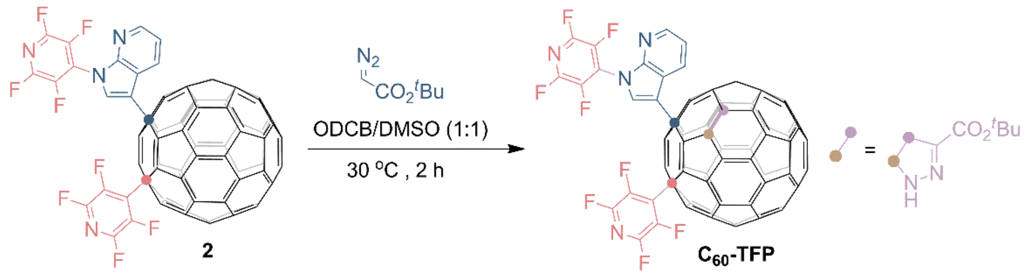

1,4-Fulleroindole **2** (29.0 mg, 0.025 mmol) and tert-butyl diazoacetate (18.0 mg, 0.125 mmol) were dissolved in ODCB (5 mL) and DMSO (5 mL) at room temperature under N_2 atmosphere. After full dissolution, the reaction solution was stirred in an oil bath at 30 °C for 2 h. Then, the mixture was cooled to room temperature and then evaporated in vacuo. The residue was separated on a silica gel column with carbon disulfide/dichloromethane/ethyl acetate (12:4:0.5) as the eluent to give C_{60}-TFP (28.1 mg, 86%) as black amorphous solid. **^{1}H NMR** (400 MHz, CS_2/d_6-DMSO) δ 10.20 (s, 1H), 8.55 (d, J = 9.2 Hz, 1H), 8.44 (d, J = 4.4 Hz, 1H), 8.33 (s, 1H), 7.28 (dd, J =8.0, 4.8 Hz, 1H), 1.42 (s, 9H); **^{19}F NMR** (376 MHz, CS_2/d_6-DMSO) δ -88.62 to -88.80 (m, 4F), -133.32 to -133.50 (m, 4F); **^{13}C NMR** (100 MHz, CS_2/d_6-DMSO) δ (ester C), 152.19, 151.01, 149.72, 149.31, 148.70, 148.68, 148.44, 148.07, 147.37, 147.29, 147.24, 146.97, 146.78, 146.65, 146.57, 146.19, 146.08, 146.01, 145.94, 145.76, 145.61, 145.29, 145.26, 144.94, 144.69, 144.61, 144.59, 144.45, 144.37, 144.33, 144.24, 144.21, 144.09, 143.88, 143.78, 143.74, 143.72, 143.61, 143.01, 142.97, 142.88, 142.58, 142.09, 141.62, 141.43, 141.31, 140.81, 139.90, 139.81, 139.55, 137.73, 135.65, 133.98 (aryl C), 130.22 (aryl C), 128.71 (aryl C), 125.55 (aryl C), 118.32 (aryl C), 117.68 (aryl C), 114.42 (aryl C), 86.86 (sp^3-C of C_{60}), 80.36 (sp^3-C of C_{60}), 69.94 (sp^3-C of C_{60}), 54.30 (sp^3-C of C_{60}), 27.43 (-CH_3). UV-vis ($CHCl_3$) λ_{max} 360, 457 nm; HRMS (MALDI-TOF-MS, DCTB as matrix, negative mode): m/z [M]- calcd for $C_{83}H_{14}F_8N_6O_2$ 1279.05; found 1279.36.

S1.3 Device Fabrication

ITO glass (15 Ω sq^{-1}) was washed by sequential sonication with detergent, deionized water, acetone, and isopropyl alcohol for 15 min. Then, the cleaned ITO substrates were treated with UV ozone for

10 min before use. MeO-2PACz (0.3 mg/mL in isopropanol) was spin-coated onto the ITO substrate at 4000 rpm for 30 s, followed by annealing at 100 °C for 10 min in nitrogen atmosphere. The substrates were cooled down to room temperature before depositing the perovskite layer. The 1.63 M perovskite precursor solutions were constructed by mixing CsI, MAI, FAI, PbI_2 in DMF: DMSO mixed solvent (4:1, volume/volume) with a chemical formula of $Cs_{0.05}MA_{0.10}FA_{0.85}PbI_3$. 15 mol% MACl was incorporated into the precursor solution to regulate the crystallization process. The PEAI was incorporated in perovskite solution (2 mg/mL) with stirring at room temperature for 1 h. 50 μL of the perovskite precursor solution was spin-coated at 1000 rpm for 10 s and 5000 rpm for 30 s onto the MeO-2PACz ITO substrate, 180 μL CB as anti-solvent was dripped quickly on the film at 10 s before the end of the last procedure and then annealed at 100 °C for 30 min in N_2 glovebox. After cooling to room temperature, the solution of PI (0.5mg/mL in isopropyl alcohol) quickly dropped on the perovskite and spin-coated at 5000 rpm for 30 s and annealed at 100 °C for 5 min. Then, a PCBM/C_{60}-TFB or C_{60}-TFP solution (20mg/mL in CB) was deposited onto the perovskite layer by spin coating at 2000 rpm for 30 s and then annealed at 100 °C for 10 min. Finally, 6 nm BCP and 100 nm silver electrode were sequentially evaporated under high vacuum ($<4\times10^{-6}$ Torr). The device area was characterized as 0.034 cm^2 by a metal shadow mask. For the anti-reflection coating, a MgF_2 layer with a thickness of 120 nm is thermally evaporated onto the backside of PSCs.

S1.4 Characterizations

^1H, ^{19}F and ^{13}C Nuclear Magnetic Resonance (NMR) spectra were recorded on a Bruker AV 400 MHz NMR spectrometer and tetramethylsilane (TMS) was used as internal standard. Mass spectra were collected on a Bruker Autoflex Speed mass spectrometer. The morphology of perovskite film was measured using field emission scanning electron microscopy (Apreo S HiVac FEI). XPS measurements were carried out on a Thermo ESCALAB 250 instrument with a monochromatized Al Kα X-ray source in vacuum. The concentration of the solution used to prepare the ETL film was reduced to 1 mg/mL. FTIR spectra were performed as KBr pellets on a TENSOR 27 spectrometer (Bruker, Germany) at room temperature. UV-vis. absorptions were measured by a UV-vis-NIR 3600 spectrometer (Shimadzu, Japan). AFM images were obtained by a XE-7 scanning probe microscope in noncontact mode (Park systems, Korea). Kelvin probe force microscopy (KPFM) and conductive atomic force microscope measurements were performed in room temperature and dark

conditions (Dimension ICON, Bruker). The steady-state, PLQY and time-resolved PL spectra were obtained by Edinburgh FLS1000 applied with an excitation wavelength of 480 nm. The ultrafast transient absorption spectrum (TAS) was measured on a pump-probe system (Helios, Ultrafast System) with the maximum time delay of ~8 ns using a motorized optical delay line under ambient conditions. The pump pulses at 400 nm were delivered by an ultrafast optical parametric amplifier excited by a regenerative amplifier, seeded with a mode-locked Ti: sapphire oscillator and pumped with a LBO laser. SCLC was measured on electron-only (ITO/SnO_2/perovskite/ETL/Ag), and measured from 0 to 2 V with a 0.02 V step size under dark conditions. *J–V* characteristics of photovoltaic devices were conducted in a N_2-filled glovebox at room temperature by using a Keithley 2400 source meter under simulated AM 1.5G illumination from a solar simulator (Enlitech, SS-F5, Taiwan). EQE measurements were carried out by a QE-R EQE system (EnLi Technology, Taiwan). Transient photocurrent (TPC) and transient photovoltage (TPV) measurements were carried out with a system excited by a 520 nm pulse laser. Contact angles were performed on an Attension Theta Optical tensiometer (Biolin Scienfitic, Sweden). Long term operation stability measurements were performed on PVLT-6001 M−16A light stability tester (Jiangsu D&R instruments) under simulated AM 1.5G irradiation (100 mW cm^{-2}) with a standard LED light source at around 55°C. The thermal stability was tracked with the device configuration of glass/ITO/ MeO-2PACz/Perovskite/ETL/BCP/Cu heating at 85 °C. The moisture stability was tracked with the device configuration of glass/ITO/ MeO-2PACz/Perovskite/ETL/BCP/Cu under RH: 30%.

S1.5 DFT Simulations

Density functional theory (DFT) calculations were performed using the Projector Augmented Wave (PAW) method, as implemented in the Vienna Ab Initio Simulation Package (VASP)[2-5]. The electronic exchange-correlation interactions were described by the Perdew–Burke–Ernzerhof (PBE) functional within the spin-polarized generalized gradient approximation (GGA)[6]. The D3 dispersion correction[7] was employed to account for van der Waals interactions between the perovskite substrate and organic molecules.

Geometry optimization and calculation of formation energies were carried out using 3-layer Pb–I-terminated $FAPbI_3$ slabs of 3×3×1 supercell, and a vacuum region of approximately 25 Å was included to separate the slabs. A 2×2×1 Γ-centered k-point grid was used, with a plane-wave energy

cutoff of 500 eV. Energy and force convergence criteria of 10^{-5} eV and 10^{-1} eV/A°, respectively, were applied. A dipole correction was used to mitigate spurious interactions between periodic images.

The formation energy E_{f} of the passivation molecules on $FAPbI_3$ was calculated using the following equation:

$$E_f = E_{mol/FAPbI3} - E_{mol} - E_{FAPbI3}$$

where $E_{mol/FAPbI3}$ is the total energy of the $FAPbI_3$ surface with the adsorbed passivation molecule per surface unit cell, E_{FAPbI3} is the energy of the clean $FAPbI_3$ surface, and E_{mol} is the energy of the isolated passivation molecule.

S2. NMR spectra

The chemical structures of **2**, C_{60}-TFB and C_{60}-TFP were characterized using a series of spectroscopic measurements, including ^{1}H, ^{13}C, and ^{19}F nuclear magnetic resonance (NMR) spectroscopy (Figures S1-S12). The ^{1}H NMR spectrum of **2** clearly shows aromatic region signals between 9.473 and 7.977ppm (Figure S1). The ^{19}F NMR spectra reveal signals ranging from -87.378 to -87.708 ppm and -134.342 to -134.517 ppm (Figure S2). Likewise, the ^{13}C NMR spectra of **2** indicate the characteristic carbon signals (Figure S3), along with an expanded ^{13}C NMR spectrum (Figure S4). The ^{1}H NMR spectrum of C_{60}-TFB clearly shows aromatic region signals ranging from 9.274 to 7.228 ppm and at 1.410 ppm (Figure S5). The ^{19}F NMR spectra reveal signals ranging from -88.988 to -89.163 ppm and from -133.895 to -135.055 ppm (Figure S6). Likewise, the ^{13}C NMR spectra of C_{60}-TFB indicate the characteristic carbon signals (Figure S7), along with an expanded ^{13}C NMR spectrum (Figure S8). The ^{1}H NMR spectrum of C_{60}-TFP clearly shows aromatic region signals at 10.201 ppm, 8.566 to 7.266 ppm, and 1.417 ppm (Figure S9). The ^{19}F NMR spectra reveal signals ranging from -88.621 to -88.795 ppm and -133.322 to -133.498 ppm (Figure S10). Likewise, the ^{13}C NMR spectra of C_{60}-TFP indicate the characteristic carbon signals (Figure S11), along with an expanded ^{13}C NMR spectrum (Figure S12).

The ^{1}H, ^{19}F, and ^{13}C NMR spectra of the compounds C_{60}-TFB and C_{60}-TFP displayed all the expected signals, which are consistent with those reported for analogous compounds.[8]

S2.1 NMR spectrum of 2

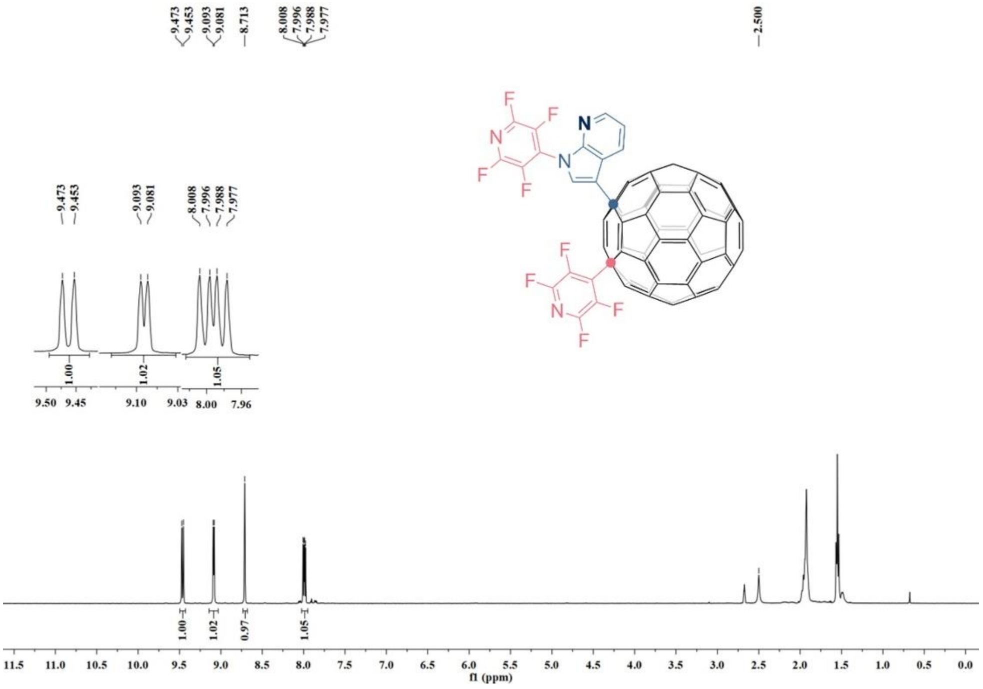

Figure S1. ^{1}H NMR (400 MHz, CS_2/d_6-acetone) spectrum of **2**.

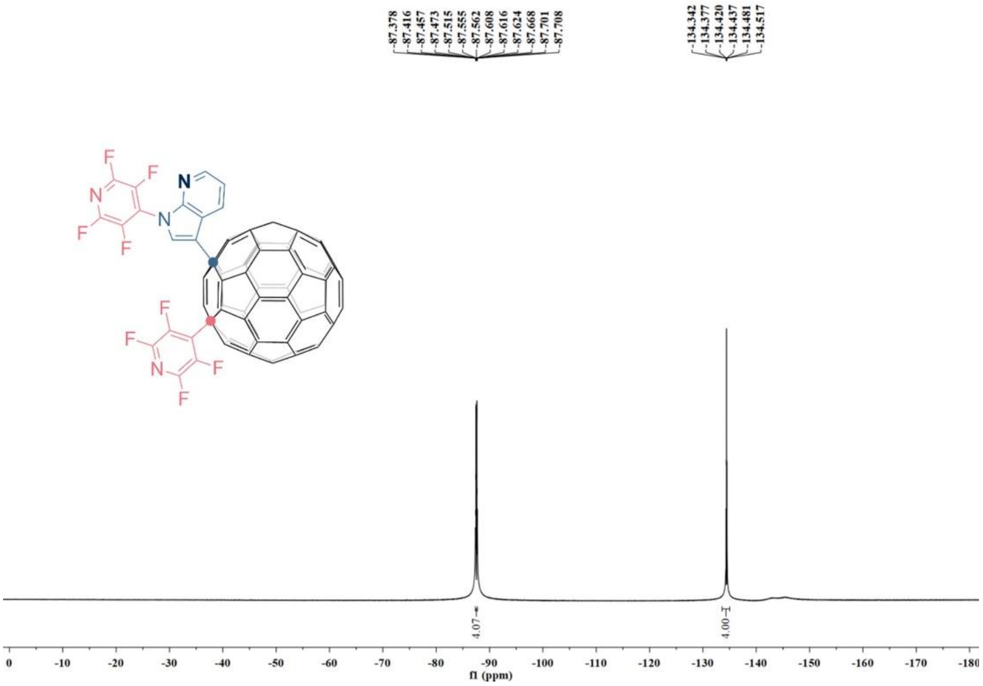

Figure S2. ^{19}F NMR (400 MHz, CS_2/d_6-acetone) spectrum of **2**.

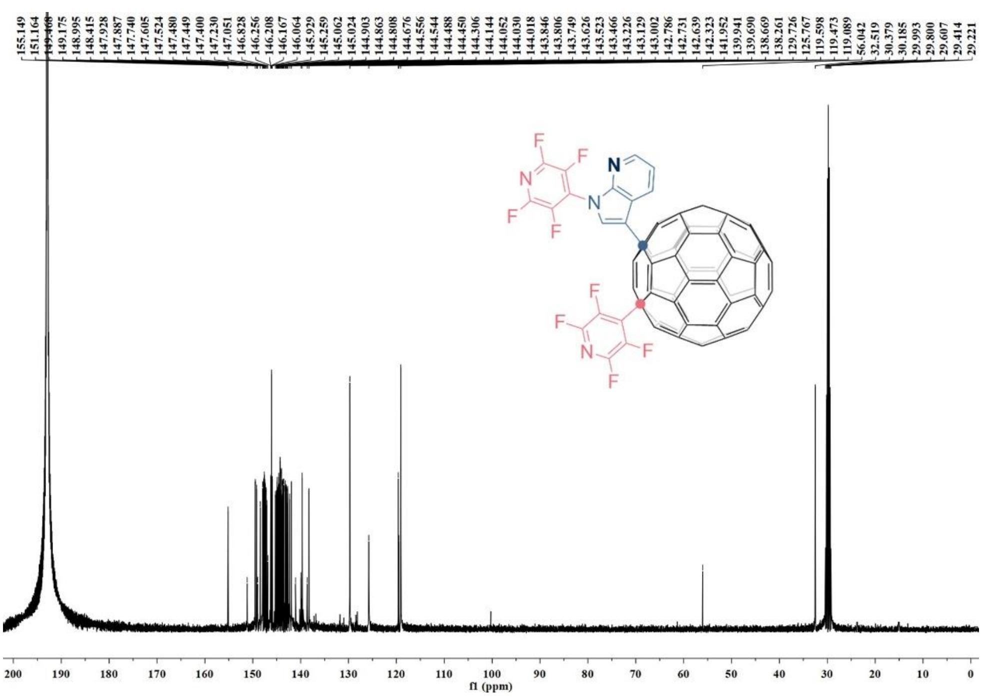

Figure S3. ^{13}C NMR (400 MHz, CS_2/d_6-acetone) spectrum of **2**.

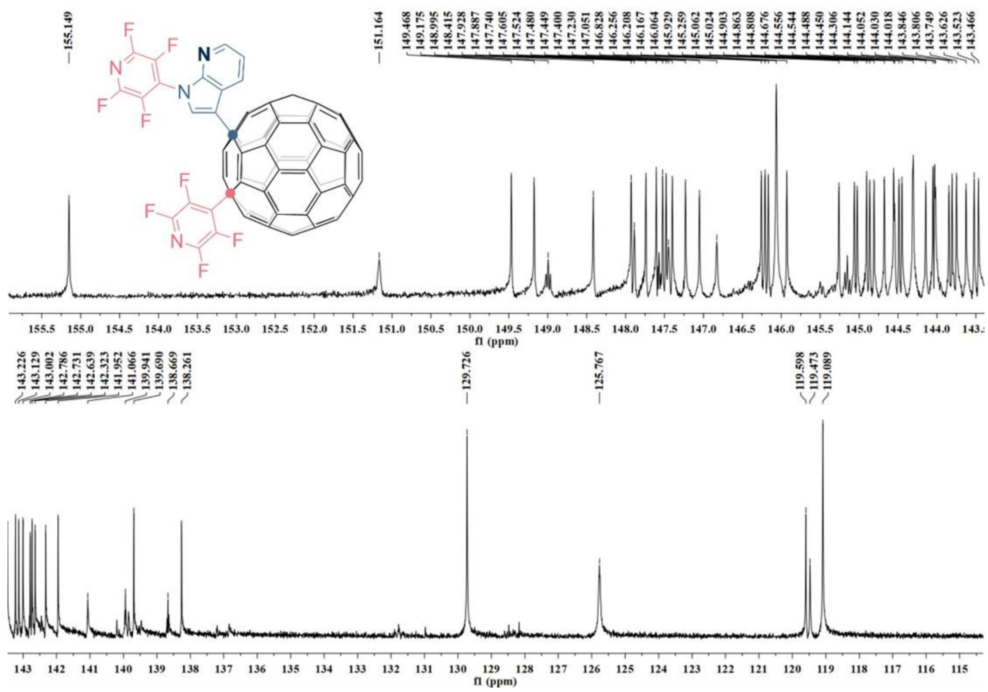

Figure S4. Expanded ^{13}C NMR (100 MHz, CS_2/d_6- acetone) spectrum of **2**.

S 2.2. NMR spectrum of C_{60}-TFB

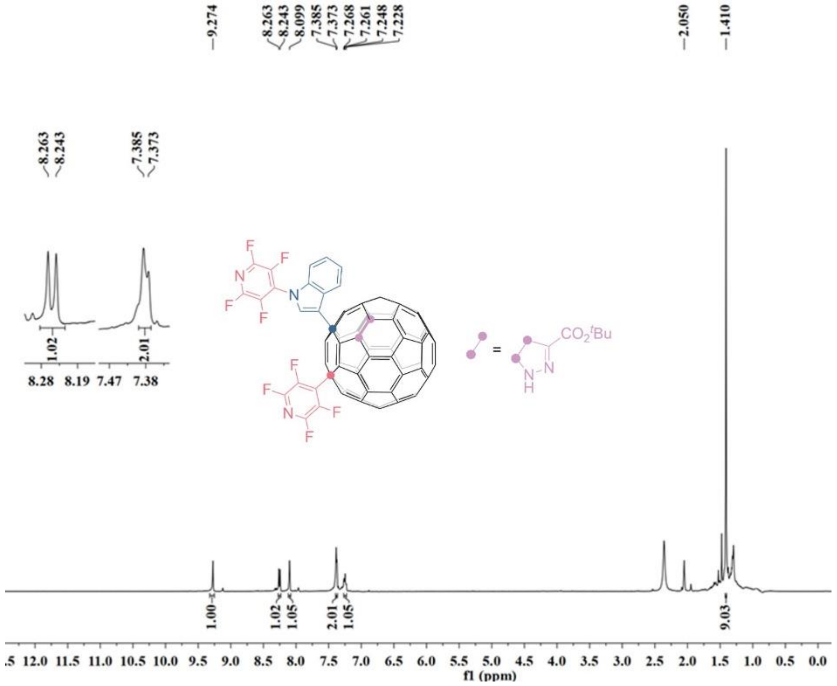

Figure S5. ^{1}H NMR (400 MHz, CS_2/d_6-acetone) spectrum of **C_{60}-TFB**.

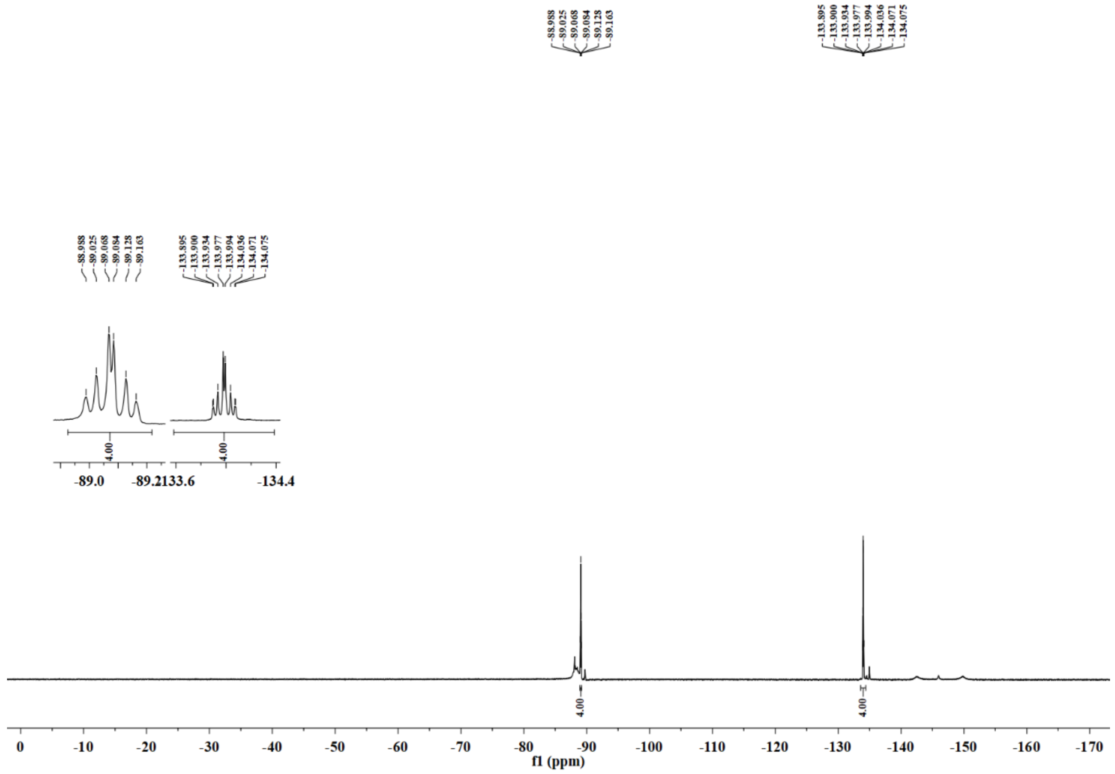

Figure S6. ^{19}F NMR (400 MHz, CS_2/d_6-acetone) spectrum of **C_{60}-TFB**.

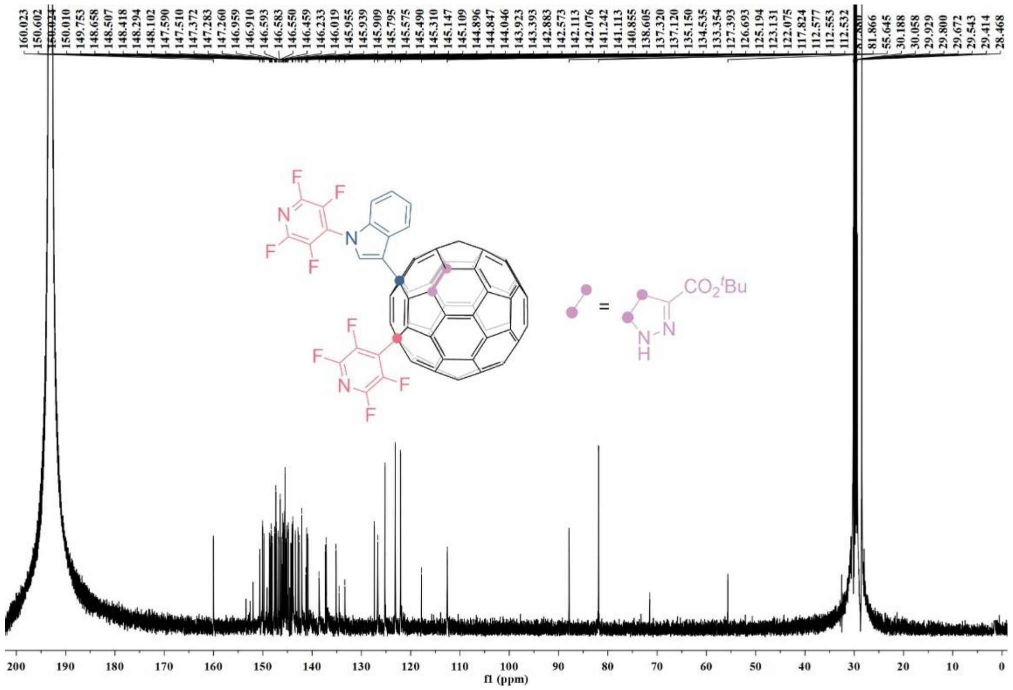

Figure S7. ^{13}C NMR (400 MHz, CS_2/d_6-acetone) spectrum of **C_{60}-TFB**.

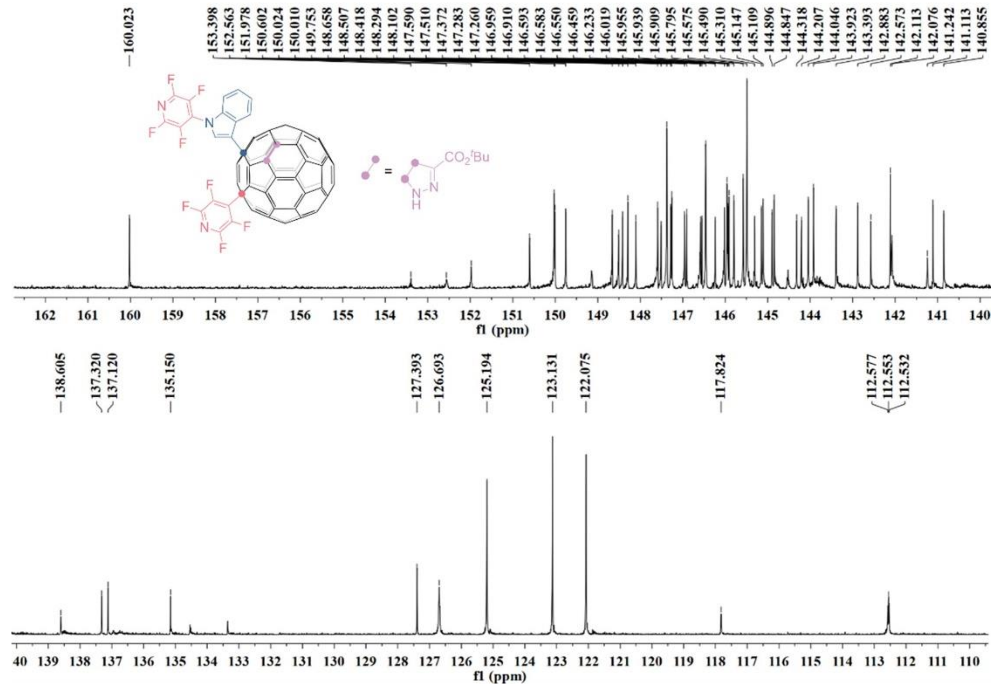

Figure S8. Expanded ^{13}C NMR (100 MHz, CS_2/d_6- acetone) spectrum of **C_{60}-TFB**.

S2.3. NMR spectrum of C₆₀-TFP

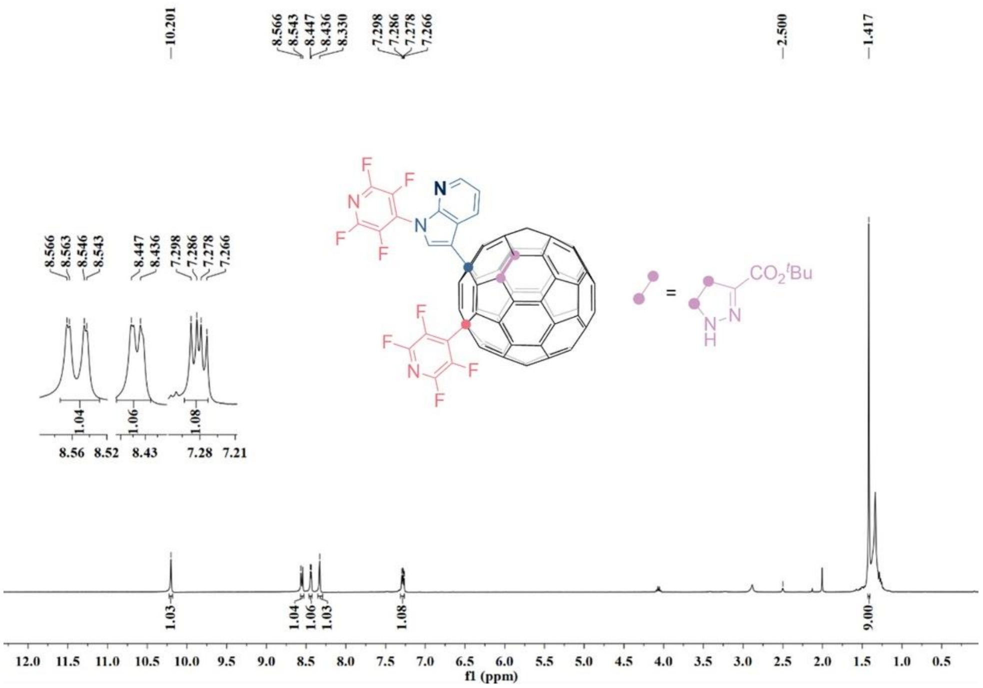

Figure S9. ^{1}H NMR (400 MHz, CS_2/d_6-acetone) spectrum of **C_{60}-TFP**.

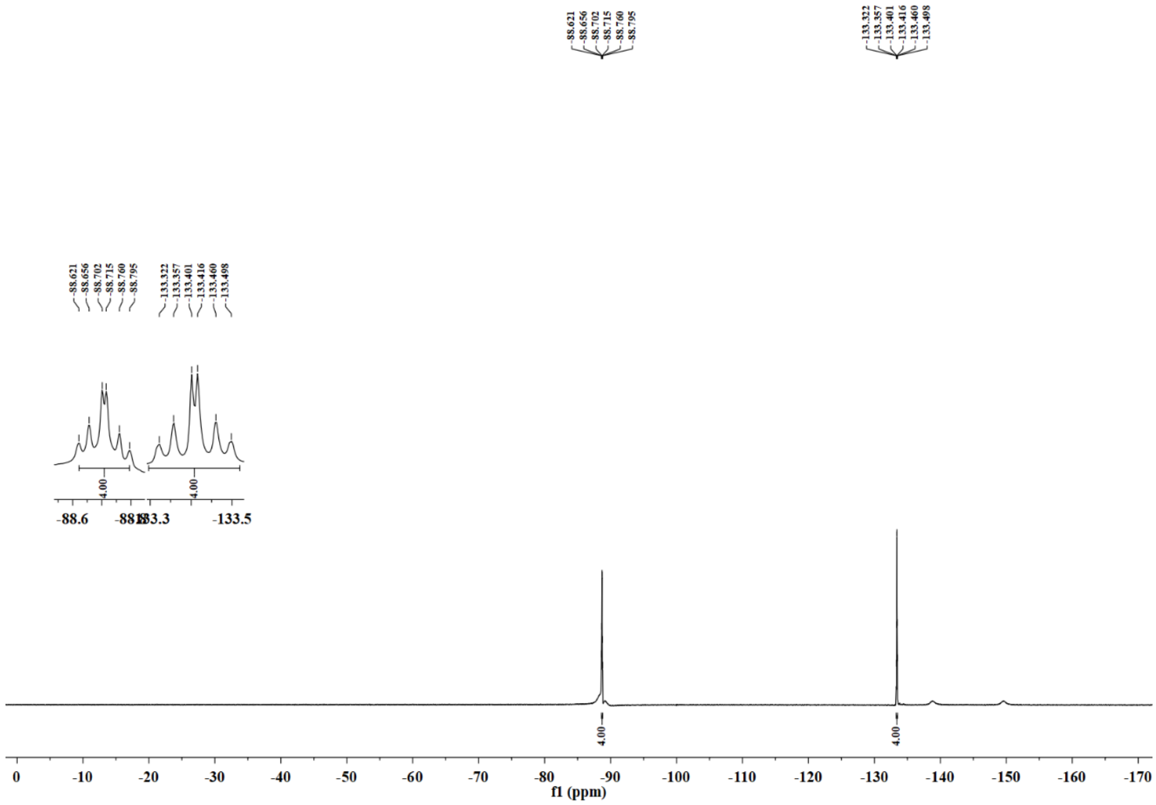

Figure S10. ^{19}F NMR (400 MHz, CS_2/d_6-acetone) spectrum of **C_{60}-TFP**.

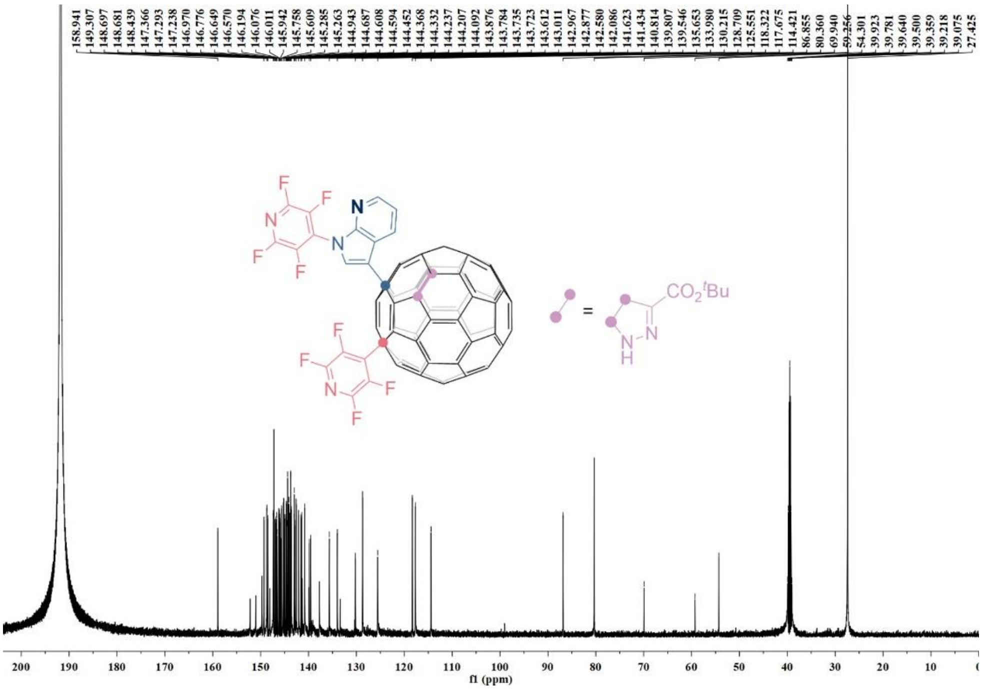

Figure S11. ^{13}C NMR (400 MHz, CS_2/d_6-acetone) spectrum of **C_{60}-TFP**.

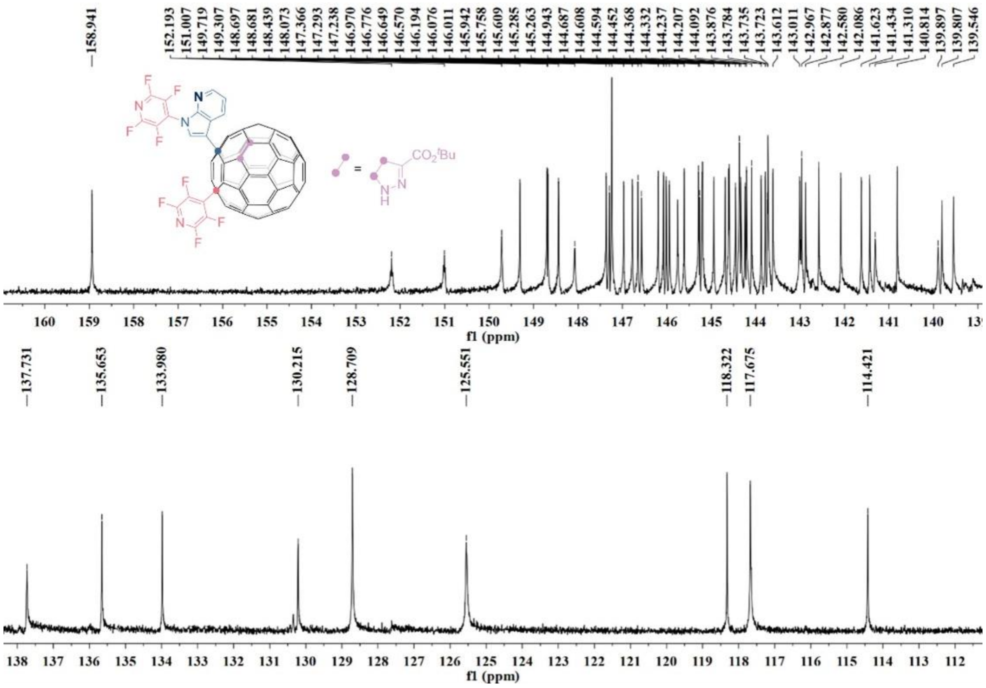

Figure S12. Expanded ^{13}C NMR (100 MHz, CS_2/d_6- acetone) spectrum of **C_{60}-TFP**.

S3. MALDI-TOF MS spectra.

Mass spectrometry (MS) analysis of C_{60}-TFB and C_{60}-TFP revealed molecular ion peaks at 1278.08 and 1279.36, respectively, which correspond to the calculated masses of $[C_{60}\text{-TFB}]^-$ (1278.06) and $[C_{60}\text{-TFP}]^-$ (1279.05).

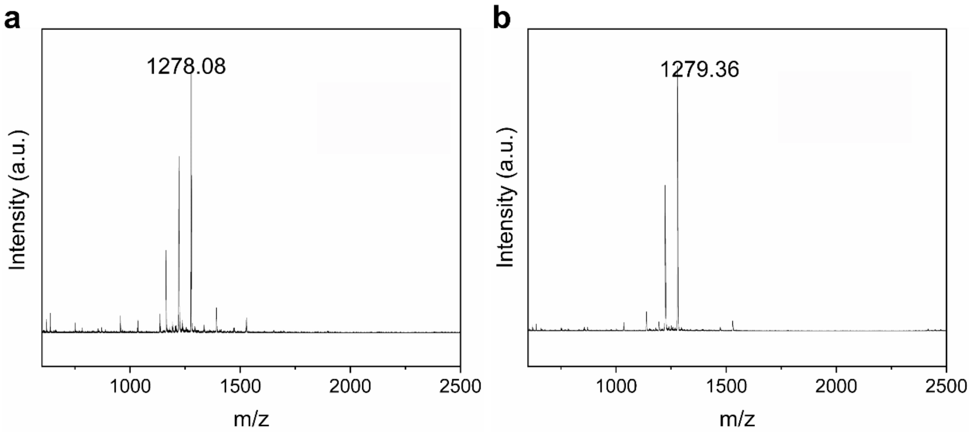

Figure S13. MALDI-TOF mass spectra of (a) C_{60}-TFB and (b) C_{60}-TFP.

S4. Electrochemical studies by Cyclic voltammetry

Cyclic voltammetry (CV) was conducted with HOKUTO DENKO HZ-7000 voltametric analyzer. Potentials in eV vs a ferrocene/ferrocenium (Fc/Fc^{+}) couple were recorded by using cyclic voltammetry in *o*-dichlorobenzene with 0.1 M tetrabutylammonium perchlorate (TBAP) as the supporting electrolyte at a scan rate of 50 mV/s. Platinum disk, platinum wire, and Ag/Ag^{+} electrodes were used as the working, counter, and reference electrodes, respectively. The estimated LUMO levels were calculated using the following equation: LUMO level = – (4.8 + E1).

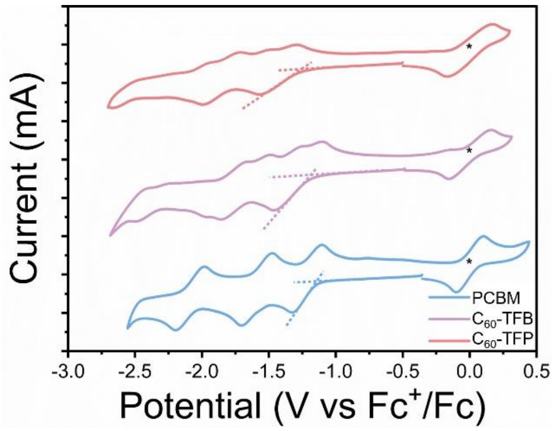

Figure S14. Cyclic voltammograms of PCBM, C_{60}-TFB, C_{60}-TFP in *o*-dichlorobenzene with 0.1 M tetrabutylammonium perchlorate (TBAP) as the supporting electrolyte at a scan rate of 50 mV/s.

S5. UV-vis spectra and the Tauc-plots

To estimate the bandgap, a V-650 spectrophotometer (JASCO Corporation) was employed to measure UV-vis spectrum. The bandgap was estimated by through calculations and the Tauc plot method. The bandgap is calculated by:

$$E_{bandgap} = \frac{1240}{\lambda_{onset}}$$

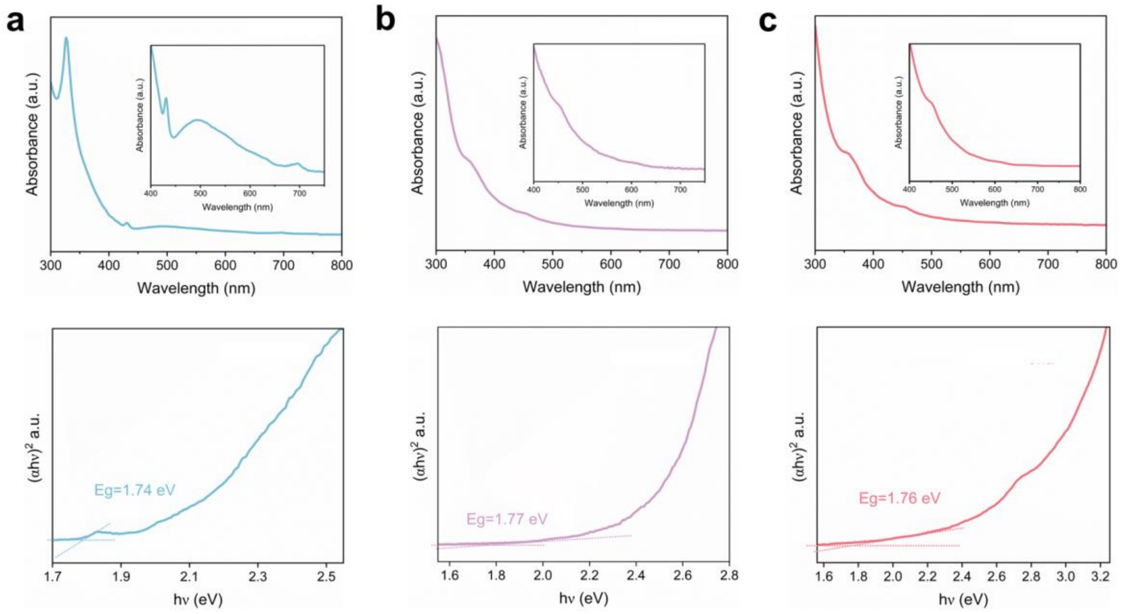

Figure S15. UV-vis spectra and the Tauc-plots of (a) PCBM, (b) C_{60}-TFB, and (c) C_{60}-TFP.

S6. C_{60}-TFB-based morphology analysis before and after aging by optical microscope, AFM and SEM with EDX

To investigate the photostability of C_{60}-TFB, we first prepared devices with C_{60}-TFB as the electron transport layer and captured images of the silver electrodes before and after 800 hours of its illumination by using an optical microscope. To visually probe the changes in C_{60}-TFB under AM 1.5G illumination, we prepared a sample consisting of ITO/MeO-2PACz/Perovskite/C_{60}-TFB and obtained AFM images before and after 1,000 hours of exposure to AM 1.5G illumination, respectively. Additionally, we obtained cross-sectional views of the devices before and after 1,000 hours of AM 1.5G illumination to investigate the changes in the distributions of Ag and I^-.

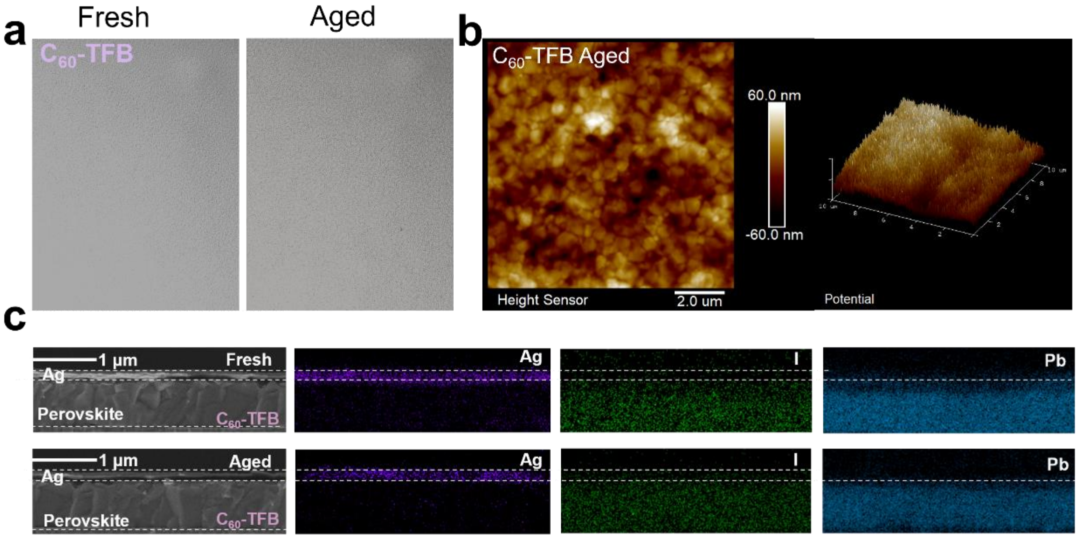

Figure S16. (a) Optical microscope photographs of Ag electrodes of devices with C_{60}-TFB, in their initial state and after continuous simulated AM 1.5G illumination aging for 800 h. (b) AFM and potential images of the C_{60}-TFB on the perovskite substrates after continuous simulated AM 1.5G illumination aging for 800 h. (c) Cross-sectional morphology of the devices in their fresh state and after 1,000 hours of continuous simulated AM 1.5G illumination aging along with EDX mapping of Ag, I, and Pb.

S7. AFM images of the fresh ETLs

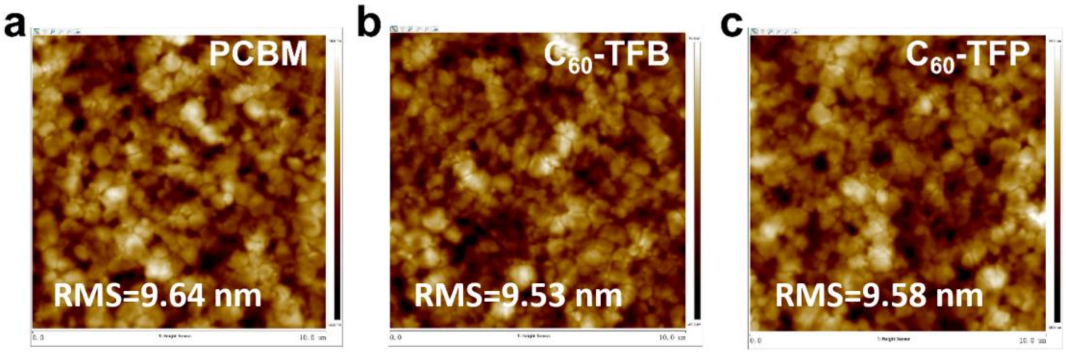

Figure S17. AFM images of the fresh (a) PCBM, (b) C_{60}-TFB, and (c) C_{60}-TFP film on the perovskite substrates.

S8. DFT results

The preferred orientation of PCBM has been reported previously.[9] Since the structures of C_{60}-TFB and C_{60}-TFP are extremely similar, for simplicity of calculation, we used DFT calculations to confirm the adsorption energies of C_{60}-TFB with different orientations on the perovskite surface, thereby confirming the preferential orientation. Then, we calculated the passivation abilities of the three molecules−PCBM, C_{60}-TFB, and C_{60}-TFP−on different types of defects on the perovskite surface, specifically under the identified preferential orientation.

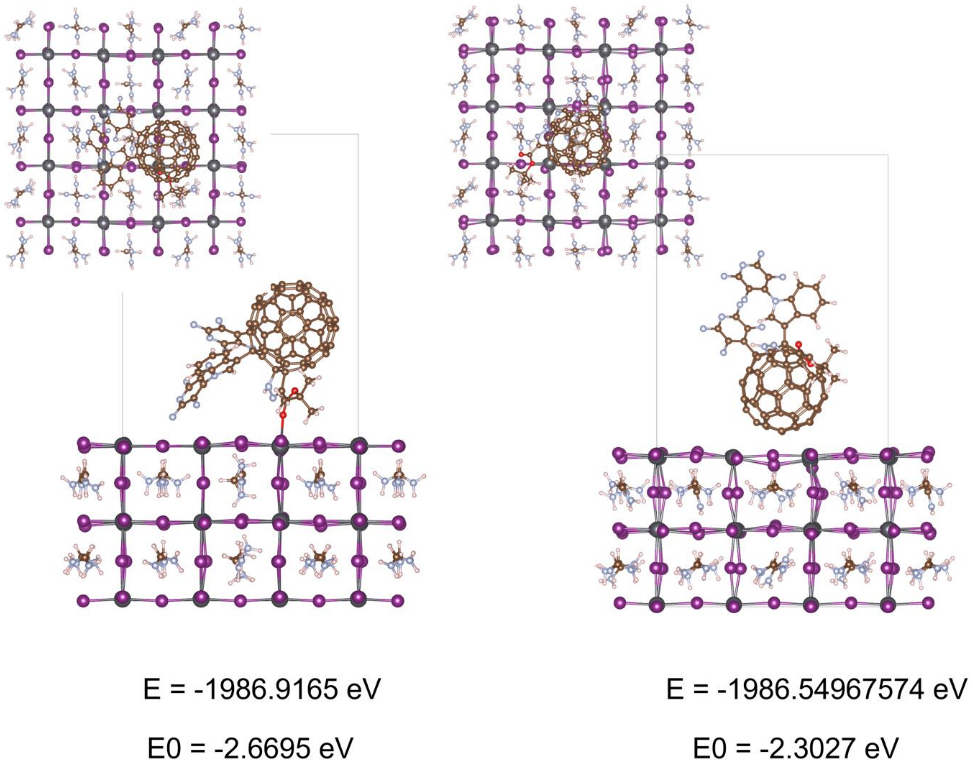

Figure S18. Theoretical calculation of perovskite covered with C_{60}-TFB of different orientations.

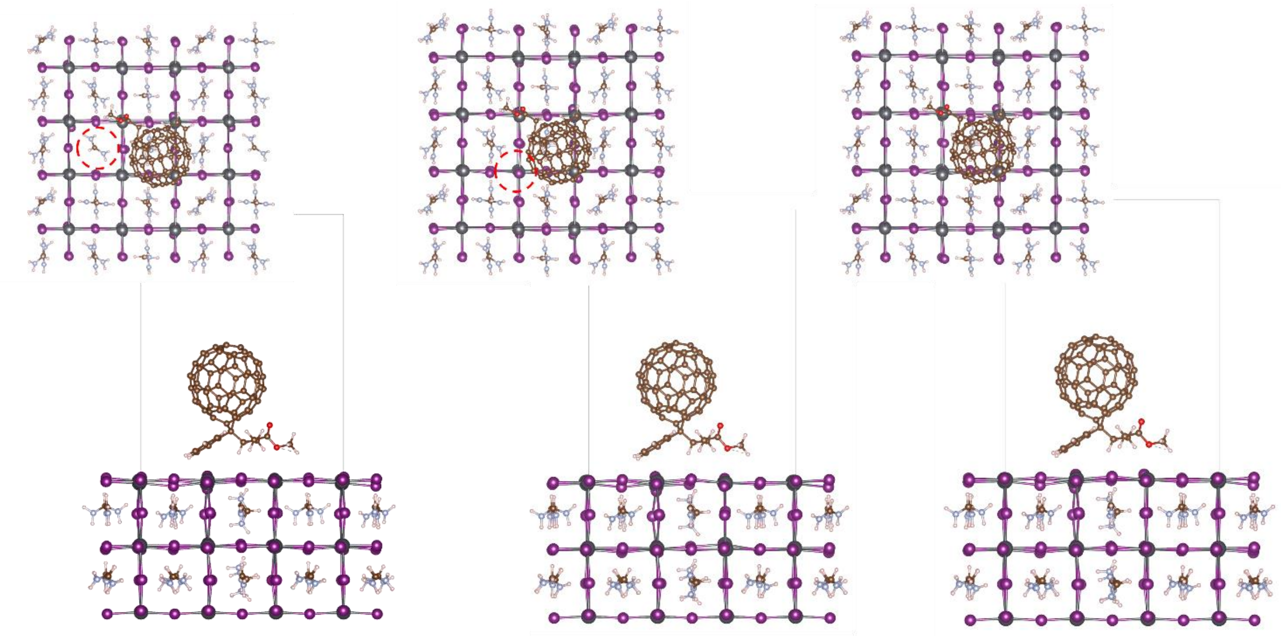

FAPbI$_3$_FA_V
E= -1756.1683 eV
Ef = -1.06643 eV

FAPbI$_3$_Pb_V
E = -1796.1340 eV
Ef = -0.9589 eV

FAPbI$_3$_I_V
E = -1799.2223 eV
Ef = -0.9038 eV

Figure S19. Absorption energy of PCBM on different types of defects.

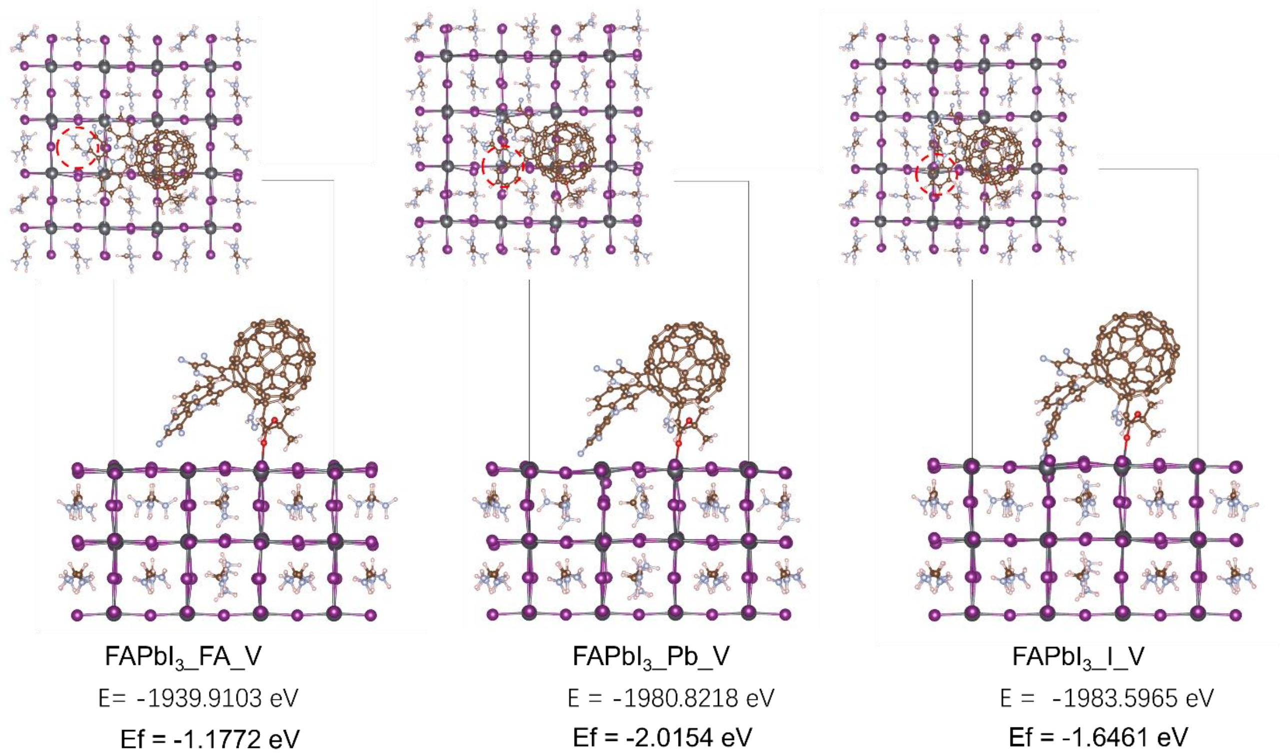

Figure S20. Absorption energy of C_{60}-TFB on different types of defects.

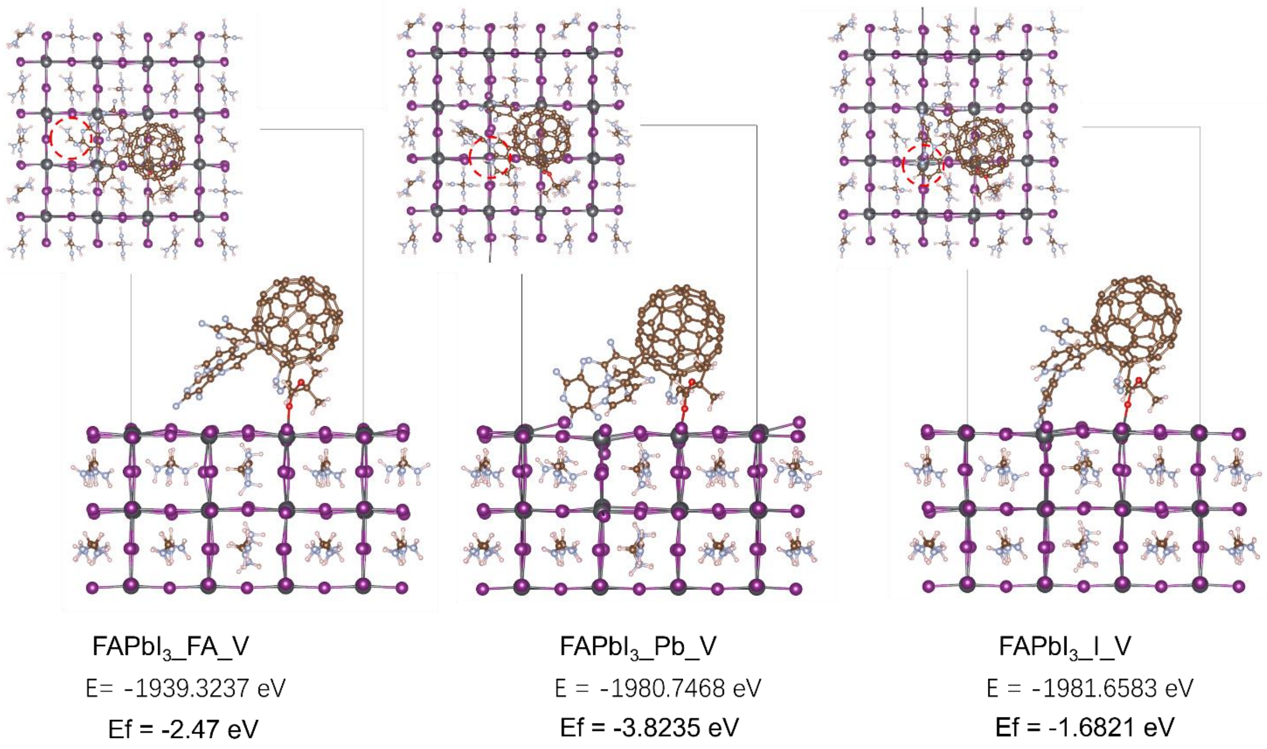

Figure S21. Absorption energy of C_{60}-TFP on different types of defects.

S9. FTIR spectra

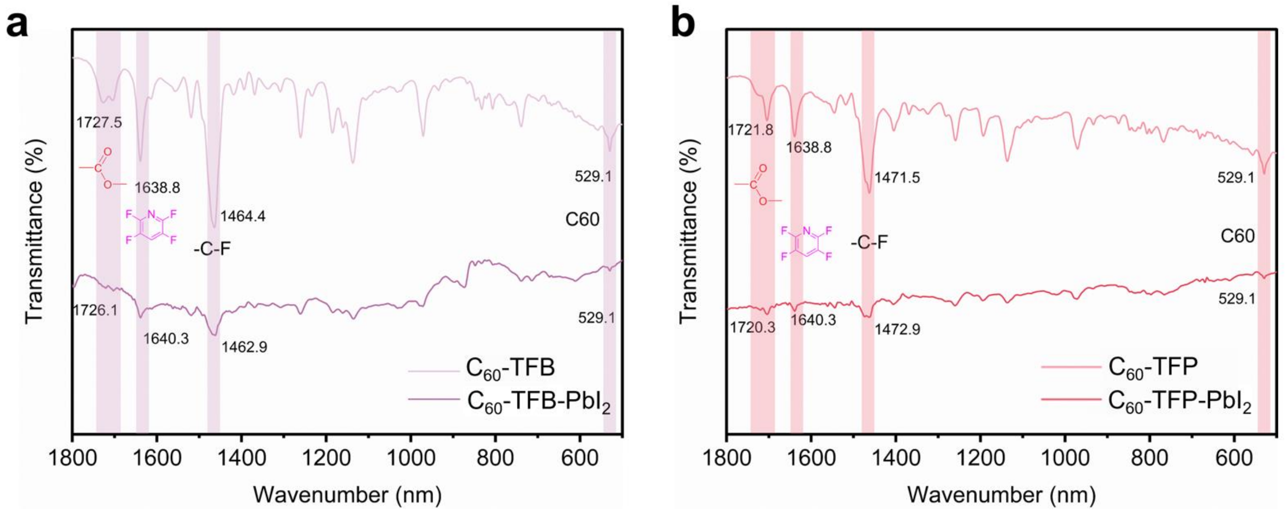

Figure S22. (a) FTIR spectra of the C_{60}-TFB and C_{60}-TFB-PbI_2. (b) FTIR spectra of the C_{60}-TFP and C_{60}-TFP- PbI_2.

S10. Valence band and conduction band analyzed by UPS spectra

To estimate the energy level of the samples, the ultraviolet photoelectron spectroscopy was employed to measure their valence band (VB) energy levels and Fermi energy levels. By combining these measurements with the previously obtained bandgap from UV-Vis spectroscopy, we can calculate the conduction band (CB) energy levels and, consequently, the overall energy level structure. The conduction band energy level can be calculated using the following formula:

$$E_{CB} - E_{VB} = E_{bandgap}$$

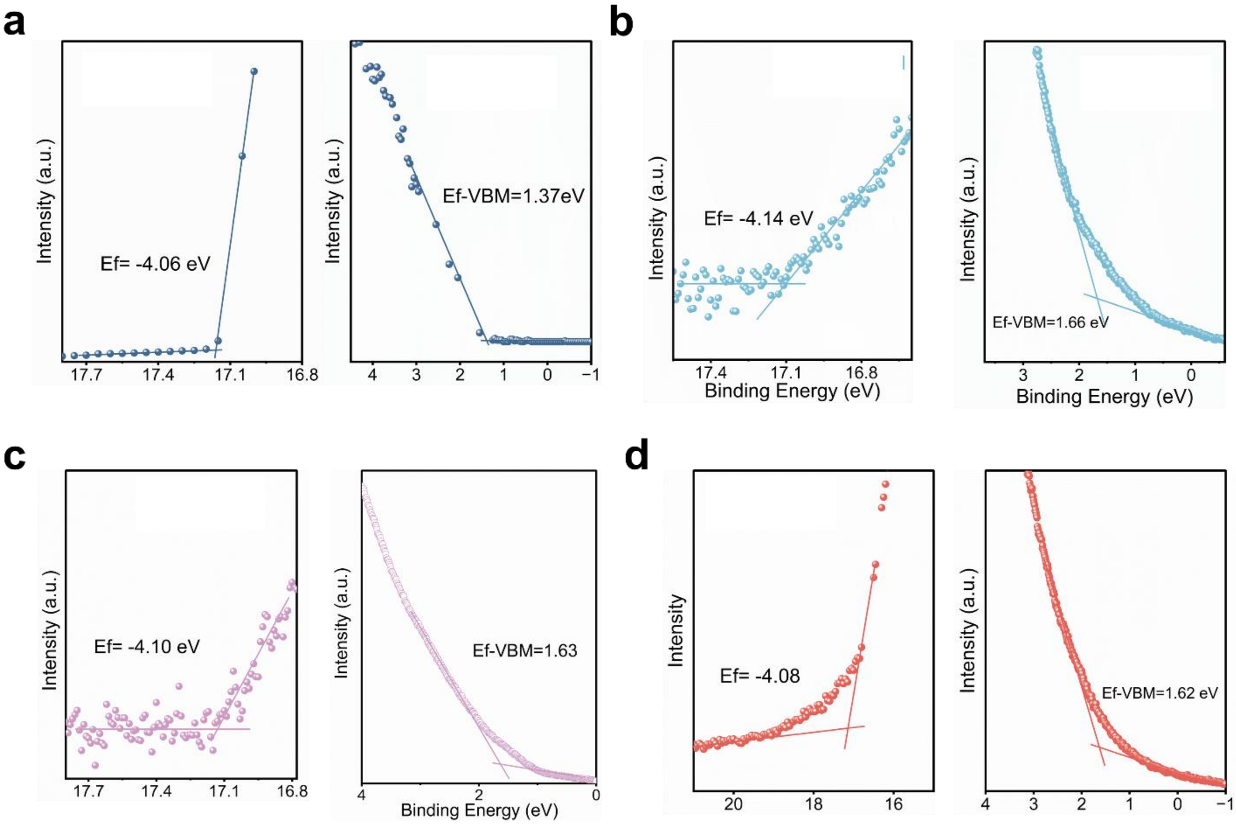

Figure S23. UPS spectra of the (a) perovskite, (b) PCBM, (c) C_{60}-TFB, and (d) C_{60}-TFP.

S11. Kelvin Probe measurement

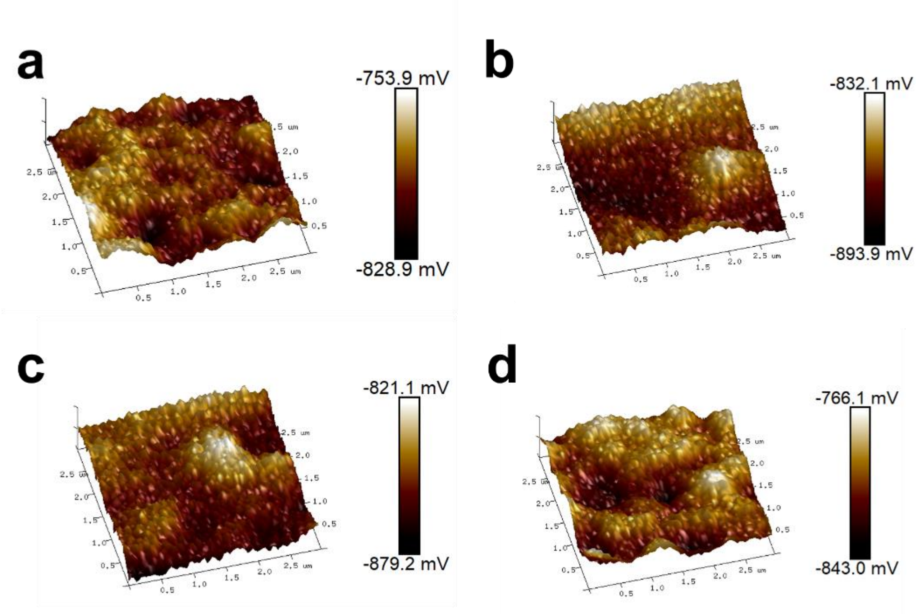

Figure S24. The KPFM of the fresh (a) perovskite film, (b) PCBM, (c) C_{60}-TFB, and (d) C_{60}-TFP.

S12. Charge carrier transport dynamics

We employ TAS and TRPL to probe the carrier transport dynamics of perovskites covered with different ETLs. Both the transient absorption kinetics and the TRPL carrier lifetime were fitted by:

$$y = y_0 + A_1 \exp(-t/\tau_1) + A_2 \exp(-t/\tau_2)$$

$$\tau_{ave} = \frac{A_1\tau_1^2 + A_2\tau_2^2}{A_1\tau_1 + A_2\tau_2}$$

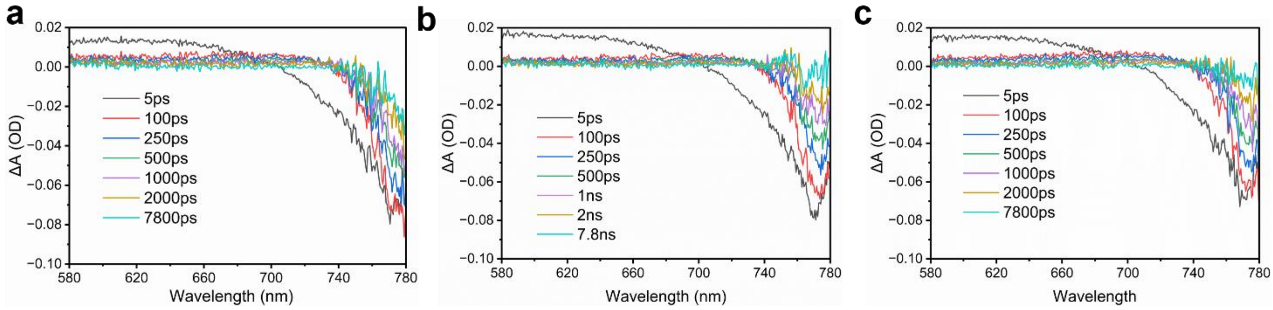

Figure S25. TAS of (a) glass/perovskite/PCBM, (b) glass/perovskite/C_{60}-TFB, and (c) glass/perovskite/C_{60}-TFP excited at 400 nm.

S13. Photovoltaic performance and stability tests

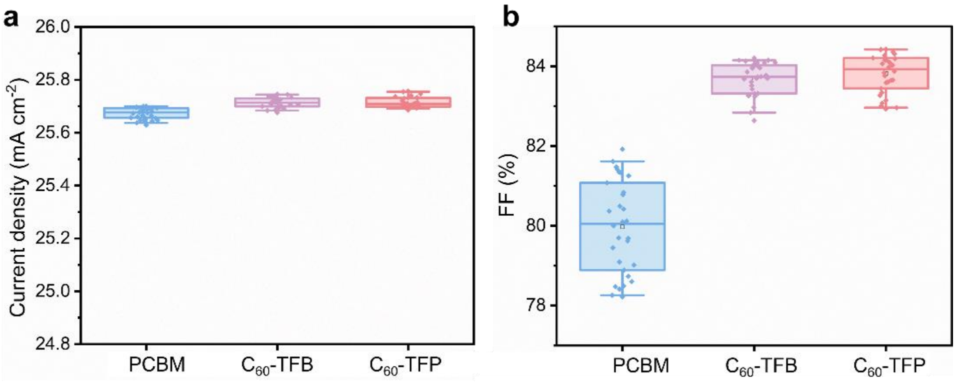

Figure S26. Statistics of J_{SC} (a) and FF (b) for PCBM, C_{60}-TFB, and C_{60}-TFP devices.

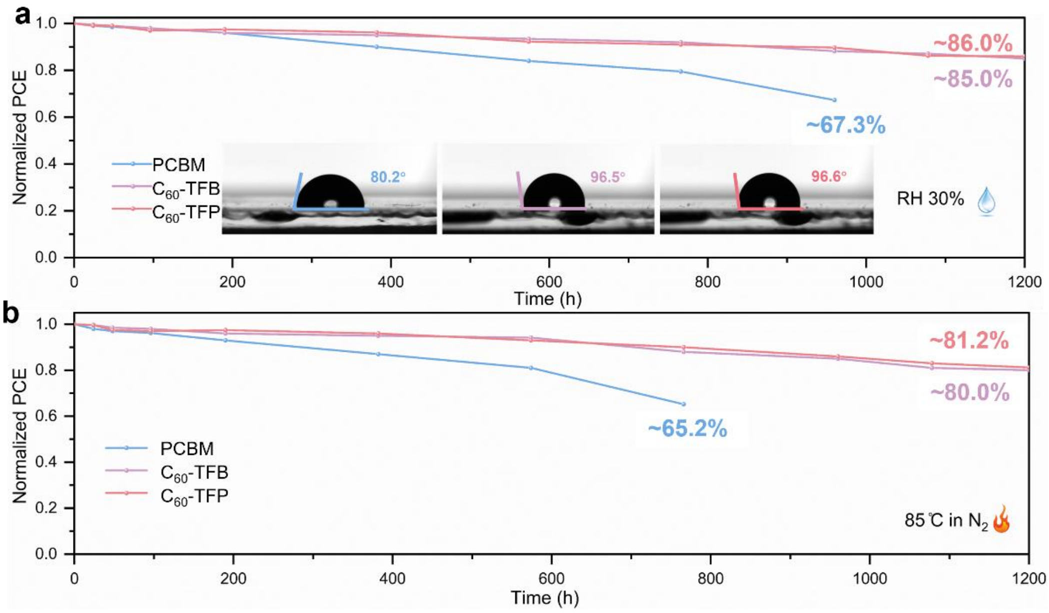

Figure S27. Normalized PCE of PCBM, C_{60}-TFB and C_{60}-TFP devices (a) under RH:30% in air condition and (b) at 85 °C in N_2 glovebox, respectively.

S14. Single-crystal X-ray crystal structure of C_{60}-TFB-AD

We attempted various methods to obtain single crystals of **C_{60}-TFB** and **C_{60}-TFP**, but were unsuccessful unfortunately. This may be attributed to the *tert*-butyl groups on **C_{60}-TFB** and **C_{60}-TFP**, which significantly enhance the solubility of the molecules and increase disorder during crystallization. Connecting adamantane groups can assist in the co-crystallization of organic small molecules,[10] thereby enabling the determination of their absolute structures through X-ray crystallography. By replacing the *tert*-butyl group in the structure of **C_{60}-TFB** with adamantane, we successfully obtained single crystals of this molecule(**C_{60}-TFB-AD)** and determined its single-crystal structure, confirming the fullerene-modified skeleton of this class of compounds.[8]

Table S1. Electrochemical data of PCBM, C_{60}-TFB, and C_{60}-TFP.

Sample	λ_{onset}^{a} (nm)	$E_{g,opt}^{b}$ (eV)	E_{red}^{onset} (eV)	E_{LUMO}^{c} (eV)	E_{HOMO}^{d} (eV)
PCBM	713	1.74	-1.15	-3.65	-5.39
C_{60}-TFB	701	1.77	-1.19	-3.61	-5.38
C_{60}-TFP	705	1.76	-1.24	-3.56	-5.32

[a] Obtained from UV-vis spectrum; [b] $E_{g,opt}$=1240/λ_{onset}; [c] E_{LUMO} =-e(E_{red}^{onset}+4.8V); [d] E_{HUMO} = E_{LUMO}- $E_{g,opt}$.

Table S2. Transient absorption fitted kinetics parameters for glass/perovskite/PCBM, glass/perovskite/C_{60}-TFB, and glass/perovskite/C_{60}-TFP at 775 nm.

Sample	A_1	τ_1 (ps)	A_2	τ_2 (ps)	τ_{ave} (ps)
PCBM	0.43	30.153	0.33	139.467	115.4401
C_{60}-TFB	0.38	21.685	0.36	154.288	137.1569
C_{60}-TFP	0.34	15.989	0.4	160.484	149.2027

Table S3. Time constants in TRPL determined by bi-exponential fittings measured on perovskite films with different ETLs.

Sample	A_1	τ_1 (ns)	A_2	τ_2 (ns)	τ_{ave} (ns)
perovskite	0.11	58.6	0.88	228	222.95
PCBM	0.13	5.82	0.85	53.11	52.33
C_{60}-TFB	0.82	1.75	0.16	56.30	48.80
C_{60}-TFP	0.93	1.28	0.11	56.70	47.82

References

[1] J. Zhang, M. Liu, W. Wang *et al.* KO^{t}Bu-Promoted, Three-Component Domino Reaction of Arenes (indoles/phenols), C_{60}, and (Per/poly) fluoroarenes: Achieving Direct C-C Cross-Coupling of Fullerene with (Per/poly) fluoroarenes. *J. Org. Chem.*, 2022, **88**, 116-131.

[2] G.Kresse and J. Hafner. *Ab initio* molecular dynamics for liquid metals. *Phys. Rev. B*, 1993, **47**, 558.

[3] P. Blöchl. Projector augmented-wave method. *Phys. Rev. B*, 1994, **50**, 17953.

[4] G. Kresse, J. Furthmüller, Efficient iterative schemes for *ab initio* total-energy calculations using a plane-wave basis set. *Phys. Rev. B*, 1996, **54**, 11169.

[5] G.Kresse and D. Joubert. From ultrasoft pseudopotentials to the projector augmented-wave method. *Phys. Rev. B* 1999, **59**, 1758.

[6] J. Perdew, K. Burke, M. Ernzerhof. Generalized gradient approximation made simple. *Phys. Rev. Lett.*, 1996, **77**, 3865.

[7] S. Grimme, J. Antony, S. Ehrlich *et al.* A consistent and accurate ab initio parametrization of density functional dispersion correction (DFT-D) for the 94 elements H-Pu. *J. Chem. Phys.*, 2010, **132**,154104.

[8] S. Yuan, M. Li, Z. Yin e*t al.* Tetrafluoropyridine Regulated Site-Selective Cycloaddition of Diazoacetates with 1,4-Unsymmetrical [60]Fullerene Adducts. *Org. Lett.*, 2025, **27**, 4457-4462.

[9] E. Hou, S. Cheng, Y. Qiu *et al.* Cross-linkable fullerene electron transport layer with internal encapsulation capability for efficient and stable inverted perovskite solar cells. *Angew. Chem. Int. Ed.*, 2025, **64**, e202416703.

[10] F. Krupp, W. Frey, C. Richert. Absolute Configuration of Small Molecules by Co-Crystallization. *Angew. Chem. Int. Ed.*, 2020, **59**, 15875-15879.
